# LIN28A‒let-7b axis drives the aggressive and proinflammatory phenotype of rheumatoid arthritis fibroblast-like synoviocytes

**DOI:** 10.1186/s13075-026-03809-7

**Published:** 2026-04-13

**Authors:** Hee Young Chae, Kyungrim Yi, Su-Geun Lim, Ji Yeong Park, Hyejin Hyung, Si-Yong Kim, Wanil Kim, Sang-Il Lee, Dong Kyu Choi, Myoung Ok Kim, Zae Young Ryoo, Jiwon Ko, Soyeon Jang

**Affiliations:** 1 School of Life Sciences, BK21 FOUR KNU Creative BioResearch Group, Kyungpook National University, Daegu, 41566 Republic of Korea; 2 Preclinical Research Center, Daegu-Gyeongbuk Medical Innovation Foundation, Daegu, 41061 Republic of Korea; 3 Core Protein Resources Center, DGIST, Daegu, Republic of Korea; 4 Department of Biochemistry, Department of Convergence Medical Science, and Institute of Medical Science, Gyeongsang National University College of Medicine, Jinju, Republic of Korea; 5 Division of Rheumatology, Department of Internal Medicine and Institute of Medical Science,, Gyeongsang National University School of Medicine, Gyeongsang National University Hospital, Jinju, Republic of Korea; 6 Department of Animal Science and Biotechnology, Research Institute for Innovative Animal Science, Kyungpook National University, Sangju, 37224 Republic of Korea; 7 Institute of Life Science and Biotechnology, Kyungpook National University, Daegu, 41566 Republic of Korea

**Keywords:** Rheumatoid arthritis, Fibroblast-like synoviocytes, LIN28A, Let-7b microRNA, Inflammation signaling, Stromal pathogenicity

## Abstract

**Background:**

Fibroblast-like synoviocytes (FLS) are central mediators of synovial inflammation and joint destruction in rheumatoid arthritis (RA). While tumor necrosis factor-α (TNFα) is known to activate FLS, the upstream regulators that connect inflammatory stimulation with sustained stromal pathogenicity remain poorly defined. The LIN28A‒let-7 microRNA axis regulates proliferation and invasiveness in diverse pathological contexts, but its role in RA FLS remains unclear.

**Methods:**

LIN28A–let-7b regulation and functional consequences were investigated in TNFα-stimulated MH7A synoviocytes and primary murine FLS. Pathway inhibitor experiments were performed using p38 and NF-κB inhibitors, and pharmacologic modulation of the LIN28–let-7 interaction was evaluated using the small-molecule inhibitor C1632. Expression of LIN28A and let-7b was also examined in synovial tissues from collagen-induced arthritis (CIA) mice.

**Results:**

TNFα stimulation induced reciprocal regulation of LIN28A and let-7b, with increased LIN28A expression and reduced let-7b levels in MH7A cells and CIA synovial tissues. LIN28A overexpression enhanced proliferation, migration, invasion, and inflammatory mediator production, and increased expression of the let-7 target HMGA2 and matrix-remodeling enzymes. These changes were accompanied by activation of MAPK and NF-κB signaling pathways. Inhibition of p38 or NF-κB attenuated LIN28A-associated inflammatory gene expression. Primary fibroblast-like synoviocytes isolated from Lin28a transgenic mice recapitulated these phenotypes. In addition, disruption of the LIN28–let-7 interaction using C1632 partially restored let-7b expression and suppressed migration, invasion, inflammatory gene expression, and signaling activation.

**Conclusion:**

LIN28A may act as an upstream regulator of synoviocyte pathogenicity in RA. Targeting the LIN28A‒let-7b axis may represent a therapeutic strategy to modulate stromal contributions to disease progression.

**Supplementary Information:**

The online version contains supplementary material available at 10.1186/s13075-026-03809-7.

## Introduction

Rheumatoid arthritis (RA) is a chronic autoimmune disorder characterized by persistent synovial inflammation, pannus formation, and progressive destruction of cartilage and bone [1–3]. Affecting ~ 1% of the global population, RA imposes a substantial socioeconomic and clinical burden [2, 3]. While RA pathogenesis involves complex interactions among innate and adaptive immune cells [1–3], increasing evidence highlights the indispensable role of resident stromal cells, particularly fibroblast-like synoviocytes (FLS), in perpetuating inflammation and driving joint damage [3, 4]. Rather than serving as passive structural components, RA FLS undergo pathological activation and acquire a tumor-like phenotype that enables cartilage invasion, immune cell recruitment, and amplification of inflammatory circuits, ultimately sustaining synovitis and bone erosion [1, 4, 5].

RA FLS display hallmark features reminiscent of transformed cells, including enhanced proliferation, apoptosis resistance, anchorage-independent growth, metabolic reprogramming, and increased migration and invasion [4, 6, 7]. These pathogenic behaviors are linked to epigenetic modifications, transcriptional reprogramming, and persistent activation of intracellular signaling cascades [8–10]. Functioning as long-lived effector cells, FLS maintain a pathogenic memory that contributes to disease chronicity even in the absence of continued immune stimulation [10, 11]. Activated FLS secrete pro-inflammatory cytokines, including interleukin (IL)-6, IL-8, IL-1β, and monocyte chemoattractant protein-1 (MCP-1), together with chemokines such as CXCL12, which recruit T cells, monocytes, and neutrophils to the synovium [12–14]. They also produce matrix metalloproteinases (MMP-1, MMP-3, and MMP-9), which degrade extracellular matrix components [15, 16], and express receptor activator of nuclear factor-κB ligand (RANKL) to promote osteoclastogenesis and bone resorption [17, 18]. Collectively, these features establish FLS as central drivers of synovial hyperplasia and joint destruction.

Tumor necrosis factor-α (TNFα) is a critical cytokine in RA, as evidenced by the clinical success of TNFα inhibitors [19, 20]. TNFα stimulation activates multiple signaling pathways in FLS, including mitogen-activated protein kinase (MAPK) cascades (ERK, JNK, p38), the nuclear factor-κB (NF-κB) pathway, and signal transducer and activator of transcription 3 (STAT3) signaling pathway [21–23]. These signaling networks induce transcriptional programs associated with proliferation, survival, cytokine and chemokine production, cytoskeletal remodeling, and matrix degradation [21, 24]. TNFα also contributes to epigenetic imprinting of FLS, reinforcing invasive and migratory characteristics [9, 25]. Despite extensive characterization of TNFα-mediated signaling, the upstream regulatory mechanisms linking inflammatory cytokine signaling to long-term phenotypic reprogramming of FLS remain incompletely defined. Identifying such regulators is essential to understanding persistent FLS activation and for developing therapeutic strategies that target stromal pathology in RA [11].

One candidate regulator of FLS pathogenicity is the LIN28‒let-7 axis [26]. LIN28A, a conserved RNA-binding protein, plays fundamental roles in embryonic development, stem cell pluripotency, organismal growth, metabolic control, and cancer progression [26–28]. It represses the maturation of let-7 family microRNAs through two mechanisms: (1) blocking Drosha-mediated processing of pro-let-7 and (2) binding pre-let-7 to inhibit Dicer-dependent maturation, thereby reducing mature let-7 levels [29]. The let-7 family regulates genes involved in cell proliferation, inflammatory signaling, and tissue remodeling, including high mobility group AT-hook 2 (HMGA2), IL-6, IL-8, KRAS, MYC, and several MMPs [30–33]. Dysregulation of the LIN28‒let-7 axis contributes to oncogenesis, fibrosis, metabolic disorders, and aberrant immune activation [27, 28, 34, 35]; however, its role in chronic inflammatory conditions such as RA remains poorly defined.

Accumulating evidence indicates that RA FLS acquire a highly invasive and tissue-destructive phenotype during chronic synovial inflammation. These pathogenic features include cytoskeletal remodeling, enhanced migratory capacity, and increased expression of matrix-degrading enzymes, enabling FLS to invade adjacent cartilage and contribute directly to joint destruction. Such phenotypic changes are driven by transcriptional reprogramming and persistent activation of intracellular signaling pathways within the inflammatory synovial microenvironment. Among the regulatory factors implicated in cellular invasion and tissue remodeling, HMGA2 has emerged as an important downstream effector of the let-7 microRNA family. HMGA2 is a chromatin-associated protein that regulates gene networks controlling cell motility, extracellular matrix remodeling, and proliferative responses [36]. In multiple pathological contexts, reduced let-7 expression leads to derepression of HMGA2, promoting cellular invasion and aggressive cellular behavior [31]. These molecular events are highly relevant to the pathogenic properties of RA FLS, which display increased migratory and matrix-degrading activity during disease progression.

Given that LIN28A is a well-established inhibitor of let-7 microRNA maturation, dysregulation of the LIN28A─let-7 axis may represent a critical upstream mechanism driving HMGA2-dependent transcriptional programs and inflammatory activation in RA synoviocytes. However, whether LIN28A contributes to the acquisition of invasive and pro-inflammatory phenotypes in RA FLS, and how this axis integrates with inflammatory signaling pathways within the rheumatoid synovium remains largely unexplored.

Recent studies suggest that the LIN28‒let-7 axis has several immunological functions, including regulation of cytokine production, macrophage activation, T cell differentiation, and immune cell metabolism [35]. Nevertheless, few investigations have examined whether LIN28A is induced in RA synovial tissue, how its expression is regulated by inflammatory stimuli such as TNFα, or whether LIN28A‒let-7 interactions contribute to FLS pathogenicity. Importantly, the mechanisms through which LIN28A influences downstream signaling pathways such as MAPK, NF-κB, and STAT3 remain unexplored in RA, representing an important gap in our understanding of stromal pathology.

Given the role of let-7 microRNAs in regulating inflammatory mediators, chemokines, MMPs, and pro-invasive factors, and the evidence linking HMGA2-dependent transcriptional programs to invasive and tissue-destructive behavior in FLS, the LIN28A‒let-7 axis emerges as a compelling candidate bridging inflammatory cytokine signaling to destructive synovial behavior. Furthermore, pharmacologic modulators of LIN28A‒let-7 interactions, such as C1632, offer potential therapeutic means to target this pathway [37, 38]. However, whether chemical modulation of the axis can attenuate FLS pathogenicity under TNFα stimulation has not yet been determined.

Based on these observations, we hypothesized that LIN28A functions as a central upstream regulator of synoviocyte pathogenicity in RA by repressing let-7b and driving downstream inflammatory and tissue-destructive programs. Because TNFα is abundant in the rheumatoid synovium and induces LIN28A expression, we used TNFα stimulation to model the inflammatory microenvironment and investigate how LIN28A reprograms FLS at the molecular and functional levels. Using human synoviocyte cell models (MH7A) and primary murine fibroblast-like synoviocytes, we examined the regulation and consequences of LIN28A‒let-7b axis and tested whether pharmacologic restoration of let-7b with C1632 could attenuate FLS activation. Collectively, our findings suggest that LIN28A may contribute to stromal pathogenicity in RA.

## Materials and methods

### Cell culture and transfection

MH7A cells were cultured in RPMI-1640 medium (Gibco, MA, USA) supplemented with 10% fetal bovine serum (FBS; Gibco) and 1% penicillin/streptomycin (P/S; Gibco), and maintained at 37 °C in 5% CO2. Stable LIN28A-overexpressing cells were generated by seeding MH7A cells into 6-well plates and transfecting after 24 h with either pCMV6-AC-GFP (Mock) or pCMV6-AC-LIN28A (LIN28A; ORIGENE, MD, USA) using Lipofectamine 3000 Transfection Reagent (Invitrogen, MA, USA), following the manufacturer’s instructions. Mock-transfected cells served as controls. Transfected cells were selected by treating with G418 sulfate (Gibco) for 14 d. LIN28A overexpression was confirmed by qRT-PCR and western blot analysis.

### Mice

Lin28a transgenic (Lin28a TG) mice used in this study were previously generated and described in detail by Jang S et al. [39]. In this transgenic line, Lin28a is constitutively overexpressed under the control of the CMV promoter. Mice were maintained under conventional conditions with a 12-h light/dark cycle and had free access to food and water. All animal procedures were approved by the Committee for Handling and Use of Animals, Kyungpook National University (approval no. KNU 2023-0097).

### Collagen-induced arthritis (CIA) mouse model

Male C57BL/6 N mice (8–10 weeks of age; *n* = 4 per group) were housed under conventional conditions with a 12 h light/dark cycle and free access to food and water. Chick type II collagen (CII; Chondrex, WA, USA) emulsified with complete Freund’s adjuvant (CFA; Chondrex) was injected subcutaneously into the tail, followed by a booster injection on day 21. On day 56, mice were euthanized and joint tissues harvested. Hind limb joints were dissected, and synovial tissues were either snap-frozen in liquid nitrogen for RNA and protein extraction or fixed in 4% paraformaldehyde for immunohistochemical analyses.

### Isolation and culture of murine fibroblast-like synoviocytes

Murine fibroblast-like synoviocytes (mFLS) were isolated from the knee and ankle joints of wild-type (WT) and Lin28a transgenic (Lin28a TG) mice. Briefly, the synovial tissue was treated with 1% type I collagenase in DMEM (Gibco) for 60 min at 37 °C. After centrifugation, the cells were resuspended in DMEM supplemented with 10% FBS and 1% penicillin-streptomycin. Cells from passage 4–6 were used for all experiments.

### RA-like inflammatory stimulation

RA-like inflammatory conditions were modeled by stimulating MH7A cells and mFLS with recombinant human or murine TNFα (10 ng/mL). For time-course analyses, cells were exposed to TNFα for 0, 6, 12, and 24 h for western blot assays; 0, 12, and 24 h for qRT-PCR analyses; and 0, 24, and 48 h for proliferation assays. For migration and invasion assays, Cells were pretreated with TNFα (10 ng/mL) for 24 h before seeding into wound healing or Matrigel invasion chambers.

### C1632 treatment

C1632 (Sigma-Aldrich, MO, USA) was dissolved in dimethyl sulfoxide (DMSO) to prepare a stock solution and stored according to the manufacturer’s instructions. To determine appropriate working concentrations of C1632, MH7A cells were initially treated with a wide range of concentrations (0, 1, 5, 10, 25, 50, 100, and 200 µM) and cell viability was assessed. Concentrations up to 50 µM did not exhibit significant cytotoxicity. Based on these results, subsequent experiments were performed using 0, 5, 10, 25, and 50 µM. Among these, 25 and 50 µM were selected for further functional and mechanistic analyses, as they significantly restored MIRLET7B expression without affecting cell viability.

For experiments performed under RA-like inflammatory conditions, MH7A cells and murine FLS (WT or Lin28a TG) were pretreated with C1632 for 2 h, then stimulated with recombinant human or murine TNFα (10 ng/mL) for 24 h. Control cells received an equivalent volume of DMSO.

### Inhibitor treatment

To evaluate the contribution of p38 MAPK and NF-κB signaling to the LIN28A-driven inflammatory phenotype, MH7A cells and mFLS were pretreated with the p38 inhibitor SB203580 (10 µM) or NF-κB inhibitor JSH-23 (10 µM) for 1 h before TNFα stimulation (10 ng/mL) for 24 h. Total RNA and protein lysates were collected for quantitative PCR and immunoblot analyses, respectively.

### Cell proliferation assay

Cell proliferation was measured using Cell Counting Kit-8 (CCK-8; Dojindo, MD, USA) according to the manufacturer’s instructions. MH7A cells (3 × 104 cells/well) were seeded in 96-well plates and allowed to attach overnight. For experiments involving TNFα stimulation, cells were treated with recombinant human TNFα (10 ng/mL) for 0, 24, or 48 h before analysis. For LIN28A expression experiments, mock-transfected or LIN28A-overexpressing MH7A cells were seeded under identical conditions, with or without TNFα stimulation.

At each time point, 10 µL of CCK-8 reagent was added per well and incubated for 1 h at 37 °C. Absorbance was measured at 450 nm using a SPECTROstar Nano (BMG LABTECH, Germany), and background absorbance from blank wells was subtracted. Relative cell proliferation was expressed as normalized absorbance values. All experiments were performed in triplicate and repeated independently at least three times.

### Cell migration and invasion assay

Cell migration was assessed using a wound healing assay. MH7A cells and mFLS were seeded in 6-well plates, grown to confluence, and pretreated with TNFα (10 ng/mL) for 24 h before the assay. A linear scratch was created across the monolayer using a sterile 200 µL pipette tip, and cellular debris was removed by gently washing with phosphate-buffered saline (PBS). Subsequently, cells were incubated in serum-reduced medium (RPMI1640 containing 1% FBS), fixed with 4% paraformaldehyde, and stained with crystal violet solution. Images were captured using a Nikon SMZ18 microscope, and migrating cells crossing the reference line were manually counted in three random microscopic fields. Wound closure (%) was calculated as follows:

$$\:Wound\:closure\:\left(\%\right)=\left(\frac{Wound\:area\:at\:0\:h-Wound\:area\:at\:24\:h}{Wound\:area\:at\:0\:h}\right)\times\:100\%$$

Cell invasion was evaluated using Matrigel-coated Transwell chambers. Inserts (8 μm pore size, 24-well; Corning, NY, USA) were coated with growth factor-reduced Matrigel (BD Biosciences, CA, USA) and polymerized at 37 °C. Cells were pretreated with TNFα (10 ng/mL) for 24 h, harvested, resuspended in serum-free medium, and seeded into the upper chamber at a density of 5 × 104 cells/insert. The lower chamber contained medium with 10% FBS as a chemoattractant, and cells were allowed to invade through the Matrigel and membrane for 24 h at 37 °C. Following incubation, non-invading cells on the upper surface of the membrane were removed with a cotton swab, while invaded cells on the lower surface were fixed with 4% paraformaldehyde and stained with crystal violet solution. Stained cells were imaged using a light microscope, and invading cells were quantified in at least five randomly selected fields per insert. Invasion was expressed as the mean number of cells per field.

### MicroRNA reverse transcription

Total RNA was extracted from cells or mouse synovial tissues using TRIzol reagent (Invitrogen, MA, USA) following the manufacturer’s instructions. To assess microRNA expression, 10 ng of total RNA were reverse-transcribed using the TaqMan MicroRNA Reverse Transcription Kit (Applied Biosystems, MA, USA) and analyzed by quantitative real-time PCR (qRT-PCR) with the TaqMan MicroRNA Assay (Applied Biosystems). Expression of let-7a, let-7b, let-7 g was measured relative to U6, and normalized values were calculated using the ∆∆Ct method.

### mRNA quantification (qRT-PCR)

Total RNA was extracted from the cells or mouse synovial tissues using TRIzol reagent (Invitrogen) following the manufacturer’s instructions. For mRNA analysis, 1,000 ng of total RNA was reverse-transcribed using the PrimeScript 1st Strand cDNA Synthesis Kit (TAKARA, Japan). Quantitative real-time PCR (qRT-PCR) was performed using TB Green Advantage qPCR premix (TAKARA) to measure the expression of human or murine mRNAs. Primers were designed using the NCBI Primer-BLAST tool and are listed in Supplementary Table 1. Relative expression levels were calculated using the ∆∆Ct method and normalized to β-actin.

### Western blot analysis

Total protein was extracted from cells and mouse synovial tissues using PRO-PREP lysis buffer (iNtRON Biotechnology, Korea) supplemented with Xpert Phosphatase Inhibitor Cocktail Solution (GenDEPOT, TX, USA). Protein concentrations were determined using a NanoDrop 2000 (Thermo Fisher Scientific, MA, USA). Proteins were resolved via sodium dodecyl sulfate–polyacrylamide gel electrophoresis (SDS-PAGE) on 10% polyacrylamide gels, transferred to polyvinylidene fluoride (PVDF) membranes (0.45 μm pore size; Millipore, Germany), and incubated overnight at 4 °C with primary antibodies: anti-LIN28A (#3978, Cell Signaling Technology, MA, USA), anti-HMGA2 (#8179, Cell Signaling Technology), anti-MMP1(#m6427, Sigma-Aldrich), anti-MMP3(#sc-6839, Santa Cruz Biotechnology), anti-phospho-p38 MAPK (Thr180/Tyr182) (#4511, Cell Signaling Technology), anti-p38 MAPK (#8690, Cell Signaling Technology), anti-Phospho-p44/42 MAPK (Erk1/2) (Thr202/Tyr204) (#9101, Cell Signaling Technology), anti-p44/42 MAPK (Erk1/2) (#4695, Cell Signaling Technology), anti-Phospho-SAPK/JNK(Thr183/Tyr185) (#9251, Cell Signaling Technology), anti-SAPK/JNK (#9252, Cell Signaling Technology), anti-Phospho-NF-kappaB p65 (Ser536) (#3033, Cell Signaling Technology), anti-NF-kappaB p65(#8242, Cell Signaling Technology), anti-Phospho-IkappaB alpha (Ser32/36) (#9246, Cell Signaling Technology), anti-IkappaB alpha (#4812, Cell Signaling Technology), anti-Phospho-Stat3 (Tyr705) (#9145, Cell Signaling Technology), anti-Stat3 (#4904, Cell Signaling Technology), and anti-beta Actin (#sc-47778, Santa Cruz Biotechnology). Subsequently, the membranes were incubated with HRP-conjugated secondary antibodies: anti-mouse IgG (#31430, Invitrogen) and anti-rabbit IgG (#31436, Invitrogen). Protein bands were visualized using West-Q Pico ECL Solution (GenDEPOT).

### Immunohistochemistry analysis

Paraffin-embedded mouse synovial tissues were sectioned at 5 µm thickness, deparaffinized in xylene, and rehydrated through a graded ethanol series. Antigen retrieval was performed in citrate buffer using microwave heating. Endogenous peroxidase activity was blocked by incubating the sections in 3% H2O2 solution for 15 min at room temperature. Sections were then blocked with 10% normal goat serum (#ab7481, Abcam) for 1 h at room temperature and incubated overnight at 4°C with rabbit anti-LIN28A Ab (#3978, Cell Signaling Technology). After three washes with PBS containing 0.1% Tween-20, slides were incubated with a biotinylated anti-rabbit IgG secondary antibody (VECTASTAIN ABC kit; Vector Labs, CA, USA). Signals were visualized using 3,3’-diaminobenzidine (DAB; Vector Labs), and nuclei were counterstained with hematoxylin. Finally, slides were dehydrated, cleared, and mounted with coverslips. Images were acquired using a MoticEasyScan One (Motic, Hong Kong).

### Immunofluorescence staining

MH7A cells were seeded onto Lab-Tek II chamber slides (2-well; Thermo Fisher Scientific) and allowed to adhere overnight. For RA-like inflammatory conditions, cells were stimulated with TNFα (10 ng/mL) for 24 h, washed with PBS, and fixed with 4% paraformaldehyde for 10 min at room temperature (25°C). Subsequently, cells were rinsed with PBS and permeabilized with 0.1% Triton X-100 in PBS for 10 min. Non-specific binding sites were blocked by incubation with 1% BSA in PBS for 30 min at room temperature. Slides were then incubated overnight at 4°C with primary antibodies against LIN28A (#sc-293120, Santa Cruz Biotechnology, TX, USA) and Ki67 (#ab16667, Abcam, UK) diluted in blocking solution. After washing three times with PBS, cells were incubated for 1 h at room temperature in the dark with Alexa Fluor 488-conjugated anti-mouse IgG Ab (#A11001, Invitrogen) and Alexa Fluor 555-conjugated anti-rabbit IgG Ab (#A21428, Invitrogen). After the final wash, slides were mounted with ProLong Gold antifade reagent containing 4’,6‑diamidino‑2‑phenylindole (DAPI; Invitrogen) and cured at 4 °C in the dark. Fluorescence images were acquired using a fluorescence microscope (DMI3000 B; Leica, Germany) under identical exposure settings across experiments.

### Statistical analysis

Data points and *n*-numbers represent individual mice or independent experiments. Statistical comparisons between two groups were performed using a two-tailed unpaired Student’s *t*-test, while multiple group comparisons were analyzed by one-way analysis of variance (ANOVA) followed by Tukey’s post hoc test. Statistical significance was defined as *p* < 0.05, with thresholds indicated as *p < 0.05, **p < 0.01, ***p < 0.001, and ****p < 0.0001. Graphs and statistical analyses were generated using GraphPad Prism Software (version 10.6.1; GraphPad Software, Inc., CA, USA).

## Results

### LIN28A–let-7b axis is dysregulated in inflammatory synoviocytes

**Fig. 1:**
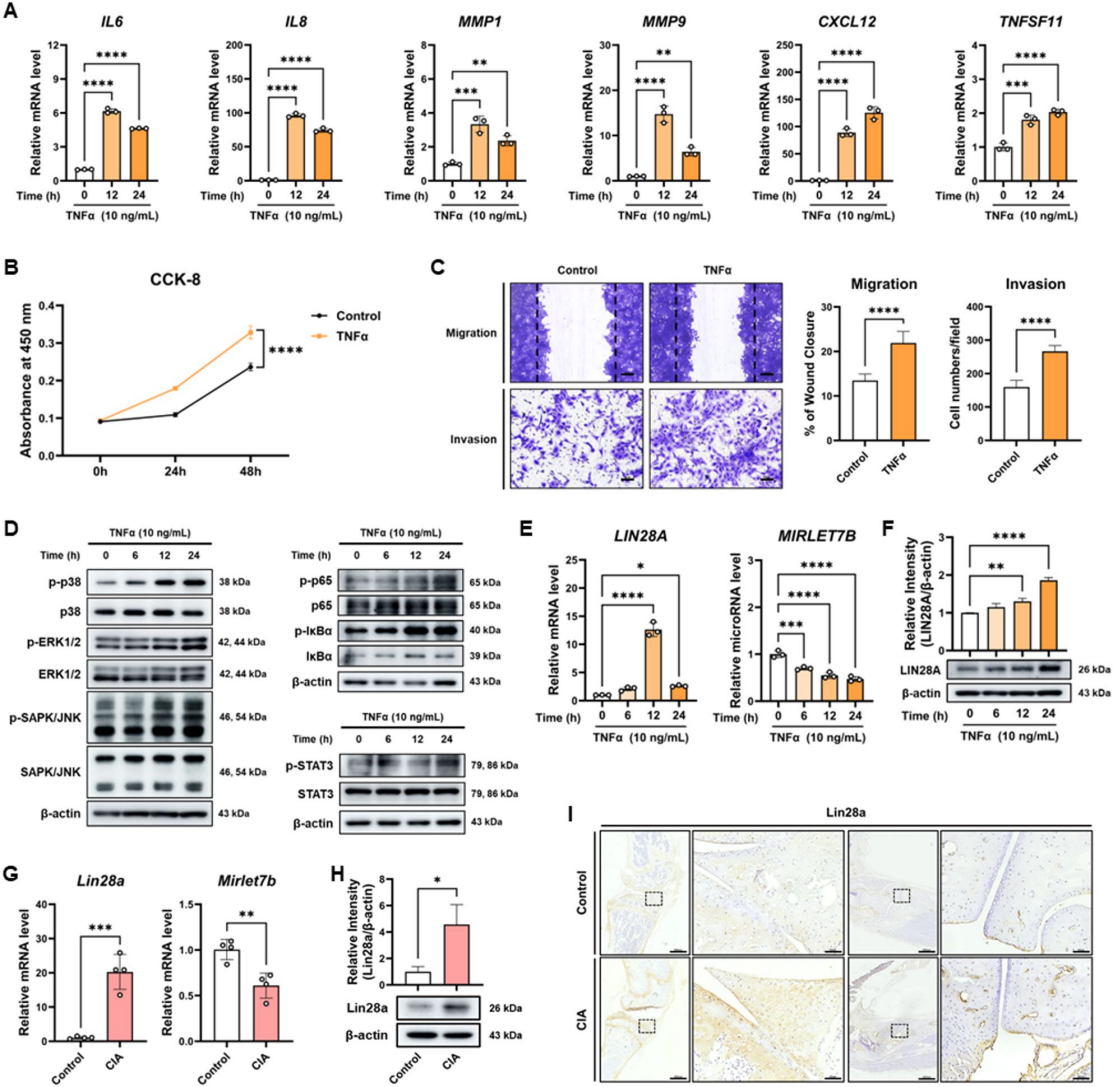
RA-like inflammatory stimulation and CIA pathology induce a characteristic LIN28A‒let-7b dysregulation pattern. **A** MH7A cells were stimulated with TNFα (10 ng/mL) for 0, 12, 24 h, and mRNA levels of IL-6, IL-8, MMP-1, MMP-9, CXCL12, and RANKL were quantified by qRT-PCR (n = 3). **B** Cell proliferation was measured by CCK-8 assay at 0, 24, and 48 h following TNFα stimulation (n = 3). **C** MH7A cells were pretreated with TNFα for 24 h prior to seeding for wound healing and Matrigel invasion assays. Scale bars: 100 μm (migration) and 60 μm (invasion). Quantification of percentage of wound closure and invading cell numbers/field were shown (n = 5). **D** Phosphorylation of p38, ERK1/2, SAPK/JNK, p65, IκBα, and STAT3 in MH7A cells stimulated with TNFα was assessed by western blot. **E** mRNA levels of LIN28A and MIRLET7B were analyzed by qRT-PCR (n = 3). **F** LIN28A protein levels were examined by western blot in MH7A cells treated with TNFα. **G** Joint tissue were collected from control and CIA mice at day 56 after primary immunization. Lin28a and Mirlet7b expression levels were quantified by qRT-PCR (n = 4). **H** Lin28a protein levels in control and CIA mice joint lysates were assessed by western blot (n = 3). **I** Synovial sections from control and CIA mice were stained for Lin28a by immunohistochemistry. Scale bar: 600 μm, 60 μm. Data are presented as mean ± SD, and statistical comparisons were performed using Student’s t-test or one-way ANOVA. *p < 0.05, **p < 0.01, ***p < 0.001, and ****p < 0.0001

To establish an inflammatory synoviocyte model relevant to rheumatoid arthritis, MH7A cells were stimulated with TNFα and the expression of inflammatory mediators and cellular phenotypes was examined. TNFα stimulation markedly increased the expression of several inflammatory and tissue-destructive genes, including IL6, IL8, MMP1, MMP9, CXCL12, and TNFSF11, as determined by qRT-PCR (Fig. 1A). In addition, TNFα treatment significantly enhanced cell proliferation, as assessed by the CCK-8 assay (Fig. 1B). Consistent with these observations, TNFα stimulation also increased the migratory and invasive capacities of MH7A cells, as demonstrated by wound healing and Matrigel invasion assays (Fig. 1C). To investigate the intracellular signaling mechanisms underlying these responses, we examined the activation of major inflammatory signaling pathways following TNFα stimulation. Immunoblot analysis revealed increased phosphorylation of p38 MAPK, ERK1/2, SAPK/JNK, NF-κB (p65), and STAT3 in TNFα-treated MH7A cells (Fig. 1D), indicating activation of multiple signaling cascades associated with inflammatory and invasive cellular responses.

Given the known regulatory relationship between LIN28A and let-7 microRNAs, we next examined whether this axis is altered under inflammatory conditions. TNFα stimulation progressively increased LIN28A expression at both the mRNA and protein levels (Fig. 1E–F). In contrast, MIRLET7B expression was significantly reduced following TNFα treatment (Fig. 1E), suggesting disruption of the LIN28A–let-7b regulatory balance in inflammatory synoviocytes. Because LIN28A regulates the maturation of multiple let-7 family members, we also examined the expression of MIRLET7A and MIRLET7G following TNFα stimulation. Both microRNAs showed modest decreases after TNFα exposure; however, the magnitude and consistency of these changes were smaller than those observed for MIRLET7B (Supplementary Fig. S1A–B). These results suggest that inflammatory stimulation preferentially affects the LIN28A–let-7b axis in synoviocytes.

To determine whether similar changes occur in vivo during inflammatory arthritis, we analyzed synovial tissues from collagen-induced arthritis (CIA) mice. Compared with control mice, CIA synovial tissues exhibited significantly elevated Lin28a expression and reduced Mirlet7b levels (Fig. 1G). Increased Lin28a expression in CIA synovium was further confirmed by western blotting and immunohistochemical staining (Fig. 1H–I).

Collectively, these findings demonstrate that TNFα induces inflammatory activation and invasive phenotypes in synoviocytes while concurrently disrupting the LIN28A–let-7b regulatory axis both in vitro and in vivo.

### LIN28A overexpression enhances aggressive phenotypes in MH7A cells

**Fig. 2:**
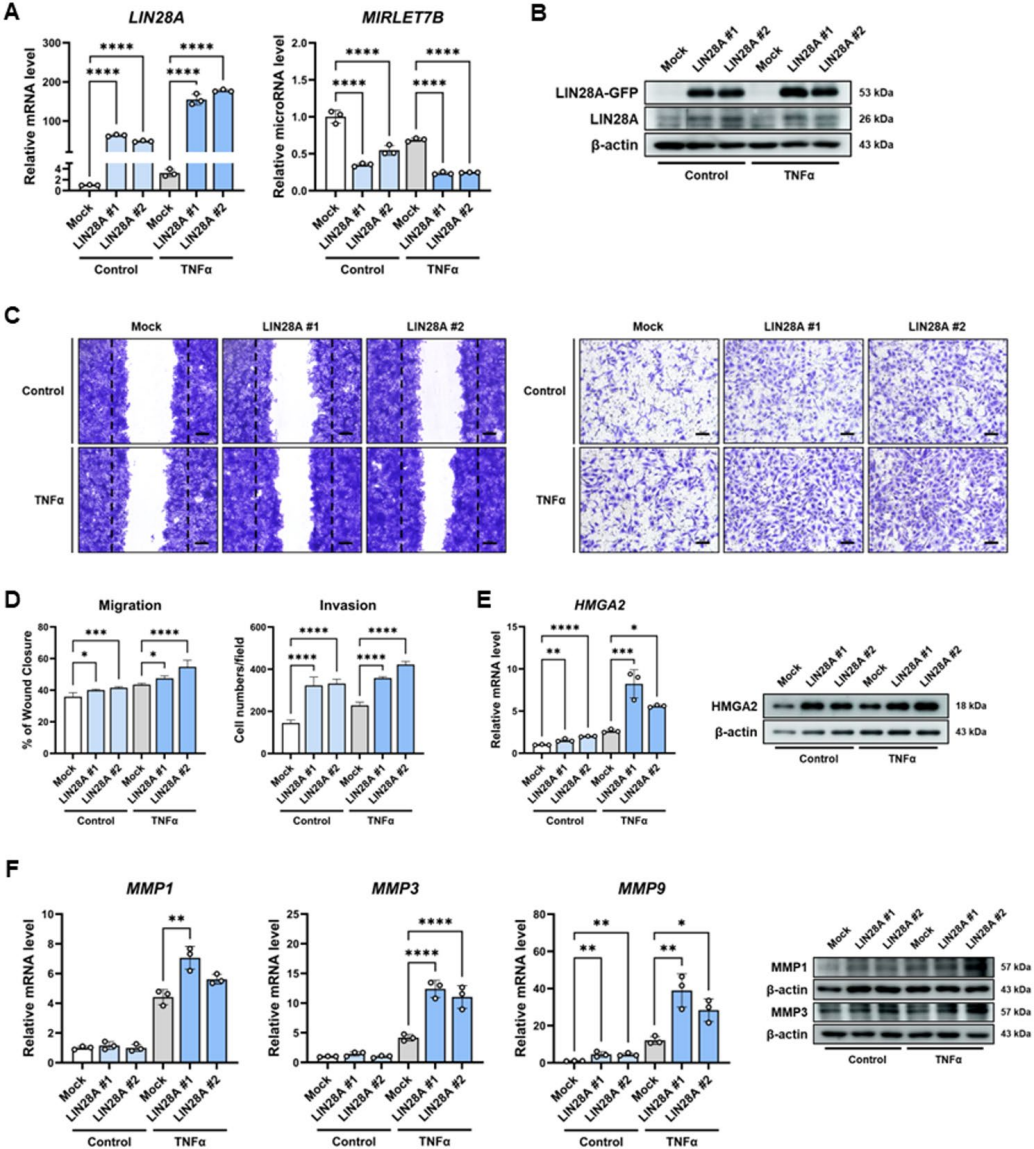
LIN28A promotes aggressive phenotype in MH7A cells. **A **MH7A cells were transfected with mock or LIN28A vector and stimulated with or without TNFα (10 ng/mL) for 24 h. LIN28A mRNA and MIRLET7B expression levels were analyzed by qRT-PCR (n = 3). **B **Immunoblot analysis of LIN28A protein expression in control and LIN28A-overexpressing MH7A cells in the presence or absence of TNFα stimulation (10 ng/mL, 24 h). **C **Representative images of wound healing and Matrigel invasion assays performed using MH7A cells pretreated with or without TNFα (10 ng/mL) for 24 h prior to seeding. Scale bars: 100 μm (migration) and 60 μm (invasion). **D **Quantification of wound closure and invading cell numbers per field (n = 5). **E **qRT-PCR and immunoblot analyses showing HMGA2 mRNA and protein levels in LIN28A-overexpressing MH7A cells stimulated with or without TNFα (n = 3). **F **qRT-PCR analysis of MMP1, MMP3, and MMP9 mRNA expression and immunoblot analysis of MMP1 and MMP3 protein levels in control and LIN28A-overexpressing MH7A cells under TNFα stimulation (10 ng/mL) for 24 h (n = 3). Data are presented as mean ± SD, and statistical comparisons were performed using one-way ANOVA. *p < 0.05, **p < 0.01, ***p < 0.001, and ****p < 0.0001

To assess the functional role of LIN28A in synoviocytes, MH7A cells were transfected with a LIN28A expression vector and stimulated with TNFα. Overexpression of LIN28A was confirmed at both the mRNA and protein levels (Fig. 2A–B). Consistent with the inhibitory effect of LIN28A on let-7 microRNA maturation, MIRLET7B expression was markedly reduced in LIN28A-overexpressing MH7A cells compared with mock-transfected controls (Fig. 2A). To determine whether other members of the let-7 family were similarly affected by LIN28A overexpression, the expression of MIRLET7A and MIRLET7G was also analyzed. Both microRNAs showed modest reductions in LIN28A-overexpressing cells; however, the degree of suppression was less pronounced than that observed for MIRLET7B (Supplementary Fig. S2A–B). These findings indicate that LIN28A overexpression preferentially suppresses let-7b among the examined let-7 family members.

The effect of LIN28A overexpression on synoviocyte proliferation was also examined. Cell proliferation was increased in LIN28A-overexpressing MH7A cells under both basal and TNFα-stimulated conditions, as assessed by the CCK-8 assay (Supplementary Fig. S3A). Consistently, immunofluorescence staining showed increased Ki67 expression in LIN28A-overexpressing cells compared with mock-transfected controls (Supplementary Fig. S3B).

The impact of LIN28A overexpression on synoviocyte phenotype was next evaluated. Wound healing and Matrigel invasion assays demonstrated that migration and invasion were significantly increased in LIN28A-overexpressing MH7A cells following TNFα stimulation (Fig. 2C–D), indicating that LIN28A is associated with enhanced aggressive cellular behavior in synoviocytes.

Because HMGA2 is a well-established target of let-7 microRNAs, its expression was examined in LIN28A-overexpressing cells. Both qRT-PCR and western blot analyses showed that HMGA2 expression was significantly increased in LIN28A-overexpressing MH7A cells (Fig. 2E). The expression of matrix-degrading enzymes associated with synoviocyte-mediated tissue destruction was further investigated. qRT-PCR analysis revealed that MMP1, MMP3, and MMP9 mRNA levels were significantly increased in LIN28A-overexpressing cells following TNFα stimulation. Consistently, western blot analysis confirmed increased protein expression of MMP1 and MMP3 (Fig. 2F). These results suggest that LIN28A may promote the expression of matrix-remodeling enzymes linked to the invasive phenotype of synoviocytes. Collectively, these findings demonstrate that LIN28A overexpression enhances the aggressive phenotype of synoviocytes and promotes HMGA2 and MMP expression under inflammatory conditions.

### LIN28A promotes inflammatory cytokine expression through p38 and NF-κB signaling in synoviocytes

**Fig. 3:**
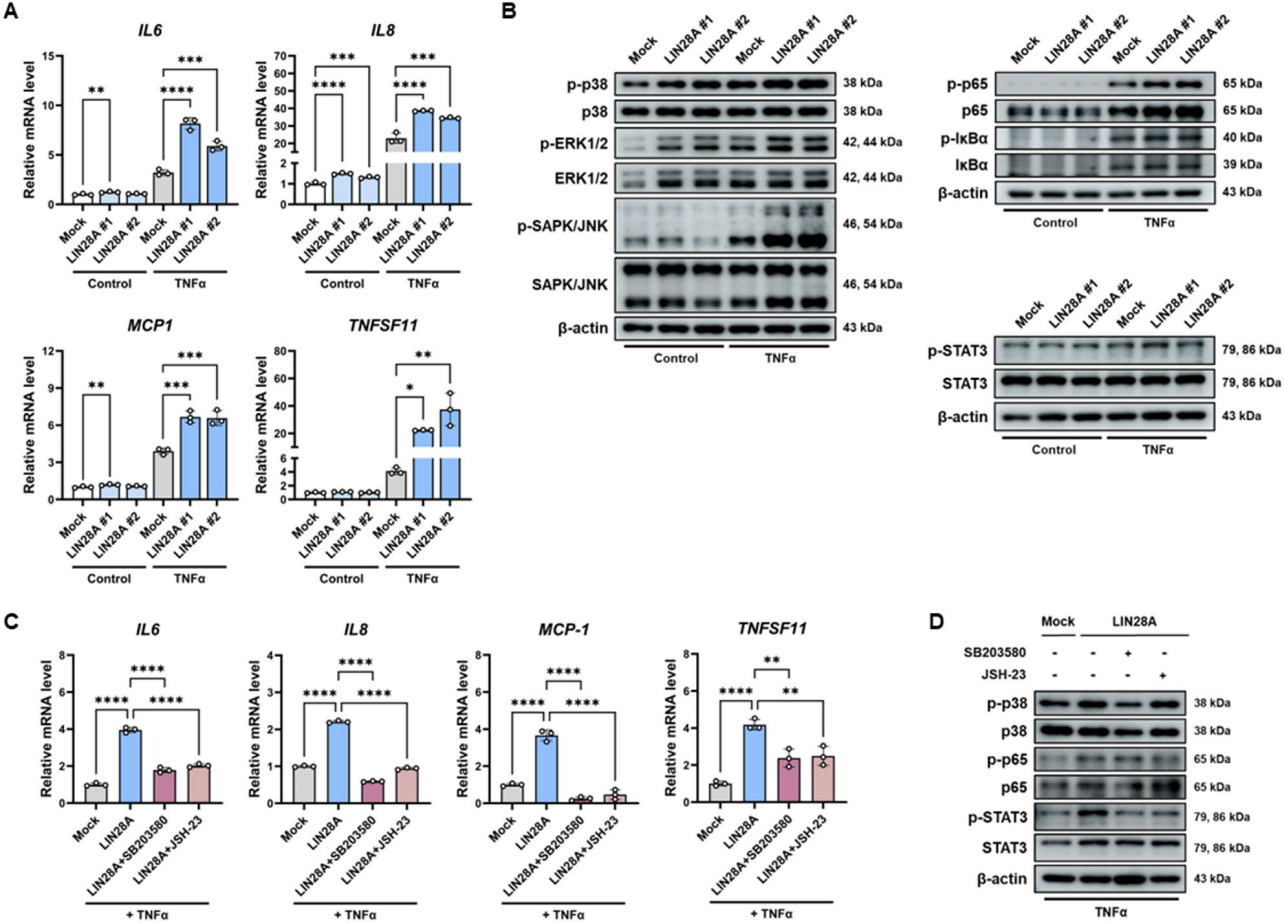
LIN28A enhances inflammatory cytokine expression through activation of p38 MAPK and NF-κB signaling in MH7A cells. **A** qRT-PCR analysis of inflammatory cytokine expression (IL6, IL8, MCP1, and TNFSF11) in control and LIN28A-overexpressing MH7A cells stimulated with TNFα (10 ng/mL) for 24 h (n = 3). **B **Immunoblot analysis of signaling pathway activation in control and LIN28A-overexpressing MH7A cells under basal conditions or after TNFα stimulation (10 ng/mL, 24 h). **C** qRT-PCR analysis of inflammatory cytokines expression in LIN28A-overexpressing MH7A cells treated with the p38 inhibitor SB203580 (10 µM) or the NF-κB inhibitor JSH-23 (10 µM). Cells were pretreated with inhibitors for 1 h followed by TNFα stimulation (10 ng/mL, 24 h) (n = 3). **D** Immunoblot analysis of p38 MAPK, NF-κB (p65), and STAT3 activation in inhibitor-treated MH7A cells following TNFα stimulation. Data are presented as mean ± SD, and statistical comparisons were performed using one-way ANOVA. *p < 0.05, **p < 0.01, ***p < 0.001, and ****p < 0.0001

To determine whether LIN28A regulates inflammatory mediator production in synoviocytes, the expression of pro-inflammatory cytokines and chemokines was examined in LIN28A-overexpressing MH7A cells following TNFα stimulation. qRT-PCR analysis showed that the expression of IL6, IL8, MCP1, CXCL12, and TNFSF11 was significantly increased in LIN28A-overexpressing cells compared with mock-transfected controls (Fig. 3A), indicating enhanced inflammatory gene expression.

The activation status of intracellular signaling pathways was next assessed. Under basal conditions, phosphorylation of p38 and ERK1/2 was increased in LIN28A-overexpressing MH7A cells. Following TNFα stimulation, LIN28A overexpression further enhanced phosphorylation of p38, ERK1/2, SAPK/JNK, NF-κB (p65), and STAT3 (Fig. 3B), suggesting that LIN28A amplifies inflammatory signaling cascades under inflammatory conditions.

To determine whether these pathways contribute to LIN28A-mediated cytokine expression, MH7A cells were treated with the p38 inhibitor SB203580 or the NF-κB inhibitor JSH-23 prior to TNFα stimulation. Inhibition of either pathway significantly reduced the expression of inflammatory cytokines and chemokines induced by LIN28A overexpression (Fig. 3C). Consistent with these findings, western blot analysis showed that SB203580 treatment reduced phosphorylation of p38 and STAT3, whereas JSH-23 treatment suppressed phosphorylation of p65 and STAT3 in TNFα-stimulated LIN28A-overexpressing MH7A cells (Fig. 3D).

Together, these results indicate that LIN28A is associated with increased inflammatory cytokine expression in synoviocytes through activation of p38 and NF-κB signaling pathways. Collectively, these results demonstrate that LIN28A may enhance inflammatory cytokine production, potentially through activation of p38 MAPK and NF-κB signaling under inflammatory conditions.

### Lin28a overexpression enhances aggressive phenotypes in primary murine FLS

**Fig. 4:**
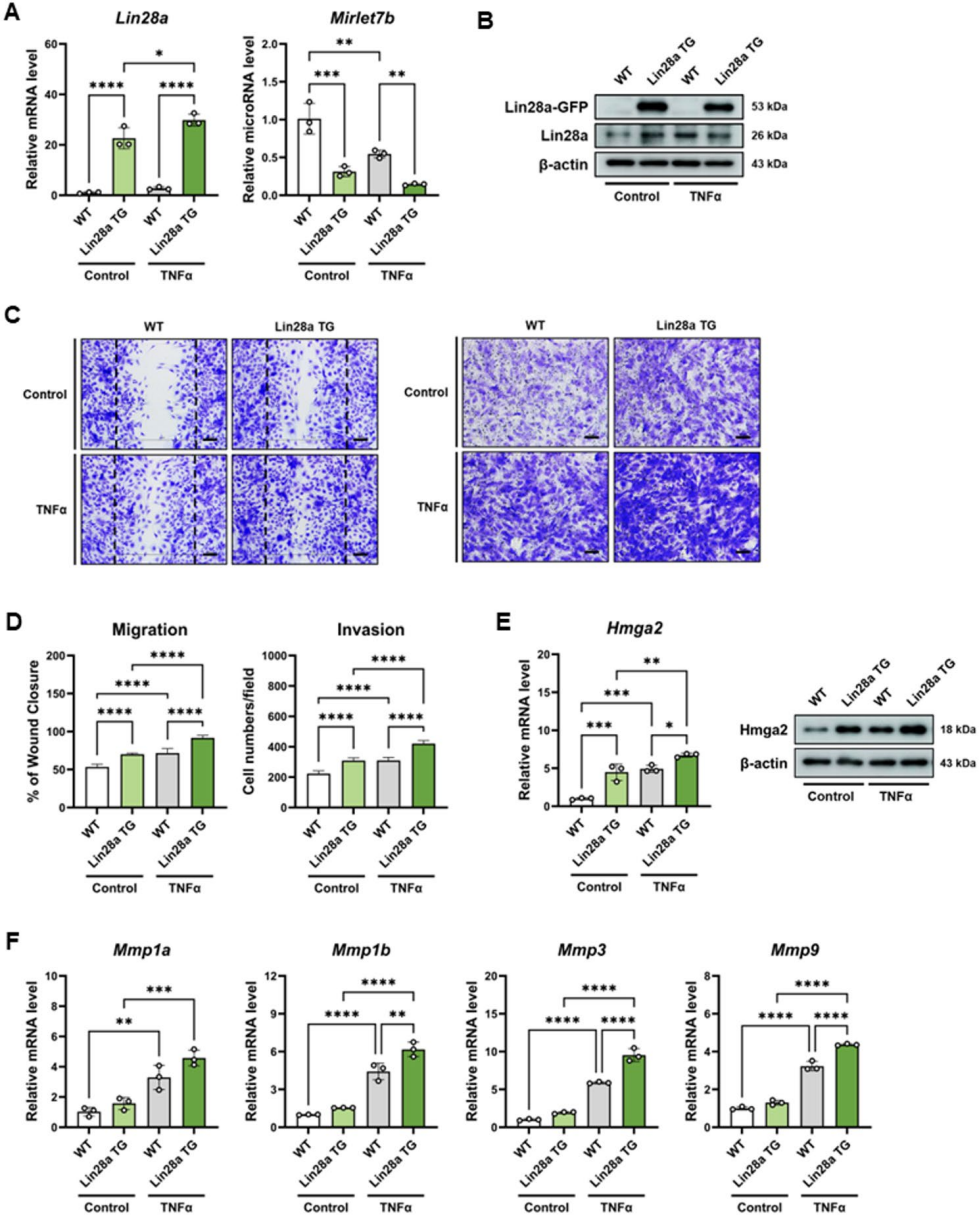
Lin28a overexpression promotes aggressive phenotypes in primary murine fibroblast-like synoviocytes. Murine fibroblast-like synoviocytes (mFLS) were isolated from wild-type (WT) and Lin28a transgenic (Lin28a TG) mice and analyzed under basal conditions or following TNFα stimulation (10 ng/mL, 24 h). **A** qRT-PCR analysis of Lin28a and Mirlet7b expression in WT and Lin28a TG mFLS under basal conditions or after TNFα stimulation (n = 3). **B** Immunoblot analysis confirming Lin28a protein expression in WT and Lin28a TG mFLS in the presence or absence of TNFα stimulation. **C** Representative images of wound healing and Matrigel invasion assays performed using WT and Lin28a TG mFLS with or without TNFα stimulation. Scale bars: 100 μm (migration) and 60 μm (invasion). **D **Quantification of wound closure and invading cell numbers per field (n = 5). **E** qRT-PCR analysis of matrix remodeling genes (Mmp1a, Mmp1b, Mmp3, and Mmp9) in WT and Lin28a mFLS following TNFα stimulation (n = 3). Data are presented as mean ± SD, and statistical comparisons were performed using one-way ANOVA. *p < 0.05, **p < 0.01, ***p < 0.001, and ****p < 0.0001

To determine whether the phenotypic effects observed in MH7A cells are reproducible in primary synoviocytes, FLS were isolated from wild-type (WT) and Lin28a transgenic (Lin28a TG) mice. Lin28a expression was markedly increased in Lin28a TG FLS, while Mirlet7b expression was correspondingly reduced compared with WT cells (Fig. 4A). Increased Lin28a protein expression in Lin28a TG FLS was further confirmed by western blot analysis (Fig. 4B).

Primary FLS derived from Lin28a TG mice displayed increased migratory and invasive capacities relative to WT FLS, as demonstrated by wound healing and Matrigel invasion assays (Fig. 4C–D). These observations indicate that Lin28a overexpression confers a more aggressive phenotype in primary synoviocytes. Consistent with the regulatory relationship between let-7 microRNAs and HMGA2, Hmga2 expression was elevated in Lin28a TG FLS compared with WT controls (Fig. 4E). Furthermore, the expression of genes associated with extracellular matrix remodeling was increased in Lin28a TG FLS. qRT-PCR analysis showed higher expression levels of Mmp1a, Mmp1b, Mmp3, and Mmp9 in Lin28a TG FLS than in WT cells (Fig. 4F).

Together, these findings demonstrate that primary FLS derived from Lin28a TG mice recapitulate the aggressive synoviocyte phenotypes observed in LIN28A-overexpressing MH7A cells.

#### p38 and NF-κB signaling contribute to Lin28a-mediated inflammatory reponses in primary murine FLS

**Fig. 5:**
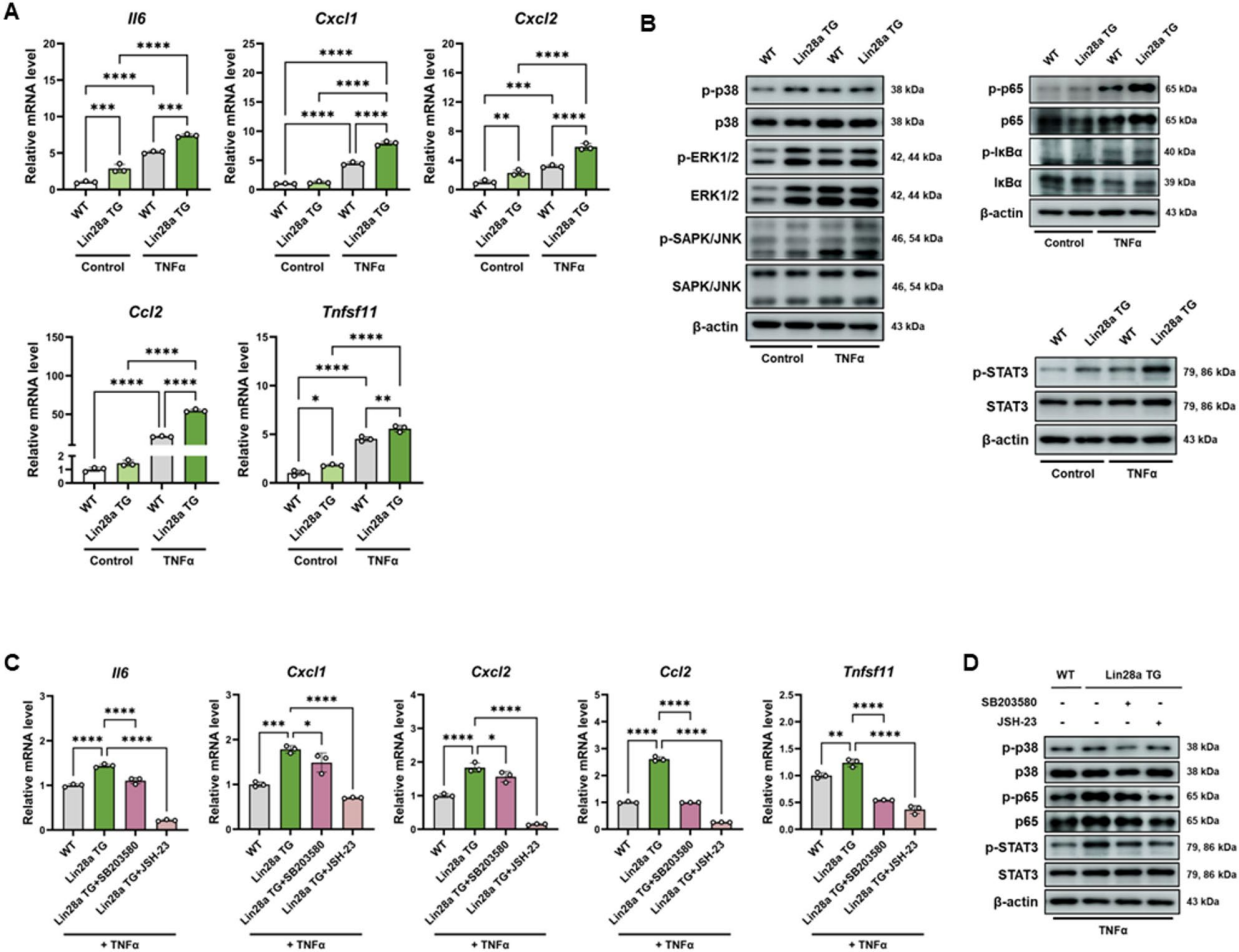
Lin28a enhances inflammatory cytokine expression through p38 MAPK and NF-κB signaling in primary murine fibroblast-like synoviocytes. **A **qRT-PCR analysis of inflammatory cytokine expression (Il6, Cxcl1, Cxcl2, Ccl2, and Tnfsf11) in WT and Lin28a TG mFLS under basal conditions or following TNFα stimulation (10 ng/mL, 24 h) (n = 3). **B **Immunoblot analysis of signaling pathway activation in WT and Lin28a TG mFLS following TNFα stimulation (10 ng/mL, 24 h). **C **qRT-PCR analysis of inflammatory cytokine expression in WT and Lin28a TG mFLS treated with p38 inhibitor SB203580 (10 µM) or the NF-κB inhibitor JSH-23 (10 µM). Cells were pretreated with inhibitors for 1 h followed by TNFα stimulation (10 ng/mL, 24 h) (n = 3). **D **Immunoblot analysis of signaling pathway activation in inhibitor-treated mFLS following TNFα stimulation. Phosphorylation levels of p38 MAPK, NF-κB (p65), and STAT3 were analyzed. Data are presented as mean ± SD, and statistical comparisons were performed using one-way ANOVA. *p < 0.05, **p < 0.01, ***p < 0.001, and ****p < 0.0001

To determine whether Lin28a overexpression influences inflammatory mediator production in primary synoviocytes, fibroblast-like synoviocytes (FLS) isolated from wild-type (WT) and Lin28a transgenic (Lin28a TG) mice were analyzed following TNFα stimulation. qRT-PCR analysis showed that the expression levels of Il6, Cxcl1, Cxcl2, Ccl2, and Tnfsf11 were significantly elevated in Lin28a TG FLS compared with WT cells (Fig. 5A), indicating that Lin28a overexpression enhances inflammatory gene expression in primary synoviocytes.

To examine the signaling pathways associated with these responses, phosphorylation of major inflammatory signaling molecules was evaluated. Under basal conditions, Lin28a TG FLS exhibited increased phosphorylation of p38, ERK1/2, and STAT3 relative to WT cells. Upon TNFα stimulation, phosphorylation of SAPK/JNK, NF-κB (p65), and STAT3 was further increased in Lin28a TG FLS (Fig. 5B).

To assess whether these signaling pathways contribute to Lin28a-associated inflammatory responses, Lin28a TG FLS were treated with the p38 inhibitor SB203580 or the NF-κB inhibitor JSH-23 prior to TNFα stimulation. Pharmacological inhibition of either pathway significantly reduced the expression of inflammatory cytokines and chemokines in Lin28a TG FLS (Fig. 5C). Consistent with these effects, western blot analysis demonstrated that SB203580 treatment reduced phosphorylation of p38, p65, and STAT3, whereas JSH-23 treatment suppressed phosphorylation of p65 and STAT3 in TNFα-stimulated Lin28a TG FLS (Fig. 5D).

Together, these findings indicate that Lin28a overexpression promotes inflammatory activation in primary synoviocytes and that p38 and NF-κB signaling pathways contribute to Lin28a-dependent cytokine expression.

### Pharmacological inhibitor of the LIN28A–let-7 interaction attenuates synoviocyte activation in primary FLS

Before examining the effects of LIN28 inhibition in primary synoviocytes, preliminary experiments were conducted in MH7A cells to establish experimental conditions. To establish appropriate experimental conditions, MH7A cells were treated with a broad range of C1632 concentrations (0–200 µM), and cell viability was assessed. No significant cytotoxicity was observed at concentrations up to 50 µM (Supplementary Fig. S4A). Based on these results, concentrations of 0, 5, 10, 25, and 50 µM were used for subsequent analyses of LIN28A and MIRLET7B expression. Notably, 25 and 50 µM significantly restored MIRLET7B expression and were therefore selected for further functional experiments (Supplementary Fig. S4B–C). In addition, C1632 attenuated TNFα-induced proliferation, migration, invasion, inflammatory gene expression, and signaling activation in MH7A cells (Supplementary Fig. S5–S7).

**Fig. 6:**
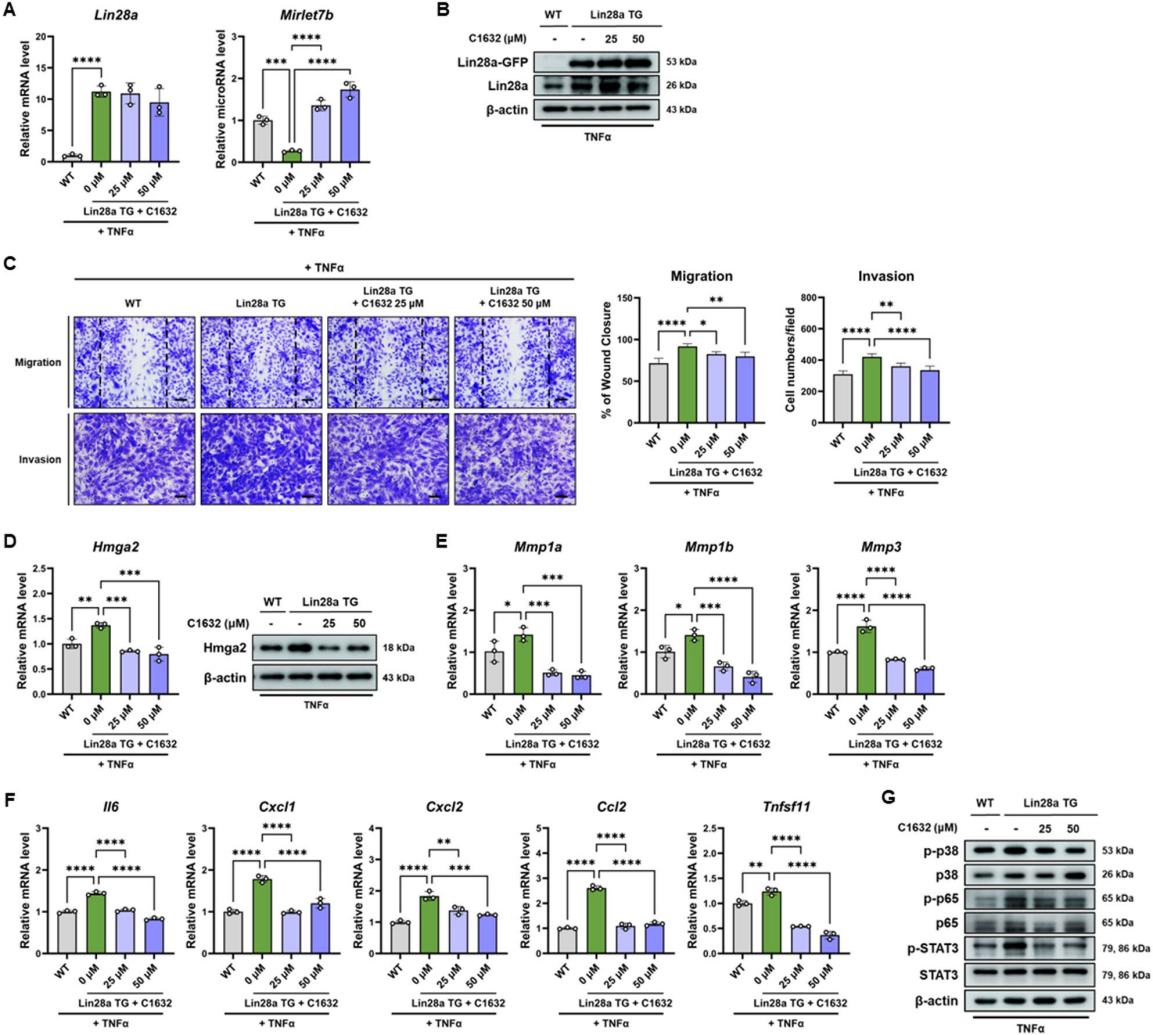
Pharmacological inhibition of Lin28a attenuates aggressive and inflammatory phenotypes in Lin28a TG mFLS. Murine FLS from WT and Lin28a TG mice were pretreated with C1632 (25 or 50 µM) for 2 h, followed by TNFα (10 ng/mL) treatment for 24 h. **A** qRT-PCR analysis of Lin28a and Mirlet7b in WT and Lin28a TG mFLS pretreated with C1632, followed by TNFα stimulation (n = 3). **B** Immunoblot analysis confirming Lin28a protein expression in WT and Lin28a TG mFLS following C1632 treatment. **C **Representative images and quantification of wound healing and Matrigel invasion assays performed using WT and Lin28a TG mFLS treated with C1632 under TNFα stimulation (n = 5). Scale bars: 100 μm (migration) and 60 μm (invasion). **D **qRT-PCR and immunoblot analyses of Hmga2 mRNA and protein expression in WT and Lin28a TG mFLS treated with C1632 under TNFα stimulation (n = 3). **E **qRT-PCR analysis of matrix remodeling genes (Mmp1a, Mmp1b, and Mmp3) in WT and Lin28a TG mFLS treated with C1632 under TNFα stimulation (n = 3). **F **qRT-PCR analysis of inflammatory cytokine expression (Il6, Cxcl1, Cxcl2, Ccl2, and Tnfsf11) in WT and Lin28a TG mFLS treated with C1632 under TNFα stimulation (n = 3). **G **Immunoblot analysis of signaling pathway activation in WT and Lin28a TG mFLS treated with C1632 following TNFα stimulation. Data are presented as mean ± SD, and statistical comparisons were performed using one-way ANOVA. *p < 0.05, **p < 0.01, ***p < 0.001, and ****p < 0.0001

To determine whether pharmacologic inhibition of the LIN28A–let-7 interaction modulates synoviocyte activation in primary cells, FLS isolated from Lin28a TG mice were treated with C1632 (25 or 50 µM) prior to TNFα stimulation (10 ug/mL). qRT-PCR analysis showed that C1632 treatment significantly increased Mirlet7b expression in Lin28a TG FLS, whereas Lin28a mRNA levels remained unchanged (Fig. 6A). Consistent with this observation, western blot analysis confirmed that Lin28a protein expression was not altered by C1632 treatment (Fig. 6B).

The functional consequences of C1632 (25 or 50 µM) treatment were next examined. Pharmacologic inhibition of the LIN28A–let-7 interaction significantly reduced the migratory and invasive capacities of Lin28a TG FLS under TNFα stimulation, as demonstrated by wound healing and Matrigel invasion assays (Fig. 6C). Consistent with the regulatory relationship between let-7 microRNAs and HMGA2, C1632 treatment markedly decreased Hmga2 expression at both the mRNA and protein levels in Lin28a TG FLS (Fig. 6D). In addition, the expression of extracellular matrix-remodeling genes, including Mmp1a, Mmp1b, and Mmp3, was significantly reduced following C1632 treatment (Fig. 6E).

C1632 treatment also attenuated inflammatory gene expression. qRT-PCR analysis revealed that the expression of Il6, Cxcl1, Cxcl2, Ccl2, and Tnfsf11 was significantly reduced in TNFα-stimulated Lin28a TG FLS (Fig. 6F). Finally, western blot analysis demonstrated that C1632 treatment reduced phosphorylation of p38, NF-κB (p65), and STAT3 in Lin28a TG FLS following TNFα stimulation (Fig. 6G).

Collectively, these findings indicate that pharmacologic inhibition of the LIN28A–let-7 interaction, without altering LIN28A expression, suppresses inflammatory signaling and pathogenic synoviocyte phenotypes.

## Discussion

RA is increasingly recognized as a disease in which stromal cells actively contribute to disease persistence and tissue destruction [4, 10, 40]. FLS acquire an aggressive and invasive phenotype that enables them to invade cartilage, promote osteoclastogenesis, and sustain inflammatory circuits independently of continuous immune cell input [4, 10]. Although inflammatory cytokines such as TNFα are well established as major drivers of FLS activation, the upstream molecular regulators that connect inflammatory cues to durable phenotypic reprogramming of synoviocytes remain incompletely defined [10, 25, 41]. In this study, we suggest that the LIN28A‒let-7b axis may represent a previously unrecognized pathway that integrates inflammatory stimulation with proliferative, invasive, and inflammatory reprogramming of FLS in RA.

Previous studies have shown that TNFα stimulation activates MAPK, NF-κB, and STAT3 signaling pathways in FLS [41, 42], leading to increased cytokine production, matrix degradation, and enhanced invasiveness [4, 10]. Our findings are consistent with this paradigm but extend it by demonstrating that TNFα stimulation is accompanied by reciprocal regulation of LIN28A and let-7b. Importantly, this pattern was observed not only in vitro but was also observed in synovial tissues from CIA mice, suggesting that dysregulation of the LIN28A‒let-7b axis is a conserved feature of arthritic pathology. This suggests that LIN28A may function upstream of canonical inflammatory signaling and implicates RNA regulatory mechanisms in sustaining FLS activation.

The LIN28‒let-7 axis has been extensively studied in developmental biology and cancer, where it governs cellular plasticity, metabolic reprogramming, and invasive behavior [26, 28]. In immune biology, emerging evidence indicates that this axis regulates cytokine production, macrophage activation, and T cell differentiation [35, 37]. Although the immunological functions of this axis have been increasingly appreciated in macrophages and lymphocytes, its role in stromal cells within chronic inflammatory tissues has remained largely unexplored. Our findings extend this regulatory paradigm to synoviocytes and suggest that dysregulation of the LIN28A–let-7 axis may contribute to pathogenic stromal activation in RA.

Functionally, LIN28A overexpression enhanced several key features associated with aggressive synoviocyte behavior, including proliferation, migration, invasion, and inflammatory mediator production. Importantly, these effects were detectable under basal conditions and were further amplified under TNFα stimulation, suggesting that LIN28A may both prime synoviocytes for pathogenic activation and increase responsiveness to inflammatory cues. These observations are consistent with the concept that stromal cells in RA acquire stable pathogenic characteristics that persist even when inflammatory stimuli fluctuate.

Mechanistically, our data implicate HMGA2 as an important downstream effector of LIN28A-mediated let-7 suppression. HMGA2 is a chromatin-associated protein that regulates transcriptional programs involved in cellular motility, cytoskeletal organization, and tissue remodeling [31]. Increased HMGA2 expression has been associated with invasive cellular behavior in multiple pathological contexts. In the present study, LIN28A overexpression increased HMGA2 expression in synoviocytes and was accompanied by upregulation of matrix-remodeling enzymes, including MMP family members. These findings support a model in which the LIN28A–let-7b–HMGA2 regulatory axis contributes to transcriptional programs that enhance the migratory and tissue-remodeling capacity of synoviocytes.

Although LIN28 proteins broadly regulate the maturation of multiple let-7 family members, our analysis indicated that let-7b showed the most consistent regulation under both inflammatory stimulation and LIN28A overexpression, suggesting a dominant contribution of the LIN28A–let-7b interaction in synoviocyte activation.

In addition to influencing invasive behavior, LIN28A also enhanced inflammatory gene expression in synoviocytes. LIN28A overexpression increased TNFα-induced expression of several inflammatory cytokines and chemokines, including IL-6, IL-8, MCP-1, and TNFSF11. These changes were accompanied by increased activation of multiple signaling pathways implicated in RA pathogenesis, including MAPK and NF-κB signaling. Importantly, pathway inhibition experiments demonstrated that pharmacologic inhibition of p38 MAPK or NF-κB significantly attenuated LIN28A-associated inflammatory gene expression. These findings suggest that LIN28A-mediated suppression of let-7b may amplify inflammatory signaling networks rather than acting solely downstream of these pathways. Notably, inhibition of either p38 MAPK or NF-κB signaling also reduced STAT3 activation, suggesting that STAT3 may function as a downstream convergence node within LIN28A-associated inflammatory signaling networks.

A notable aspect of this study is the validation of these findings in primary murine fibroblast-like synoviocytes. FLS derived from Lin28a transgenic mice exhibited enhanced migration, invasion, inflammatory gene expression, and activation of inflammatory signaling pathways compared with wild-type cells. These observations indicate that the effects of LIN28A are not restricted to an immortalized synoviocyte cell line and support the biological relevance of this pathway in primary stromal cells.

Furthermore, pharmacologic modulation of the LIN28A–let-7 axis using C1632 partially restored let-7b expression and attenuated pathogenic synoviocyte phenotypes, including migration, invasion, inflammatory gene expression, and signaling pathway activation. Unlike genetic manipulation, C1632 interferes with the interaction between LIN28 and let-7 precursors without altering LIN28A expression itself, providing a useful tool to probe the functional contribution of let-7 repression [38]. These results provide preliminary evidence that pharmacologic modulation of RNA regulatory circuits may represent a potential strategy for modulating stromal cell pathogenicity in RA.

Despite these findings, several limitations should be considered. First, although primary murine FLS were included, many mechanistic experiments relied on the MH7A cell line, and primary human RA FLS were not examined, which may limit the direct clinical relevance of the findings. Second, while dysregulation of the LIN28A–let-7 axis was observed in CIA synovial tissues, its functional contribution to disease progression was not directly evaluated in vivo, and thus causality cannot be established. Third, pharmacologic inhibition using C1632 provides indirect evidence and may be subject to off-target effects, which should be considered when interpreting these results. Finally, given the complexity of LIN28A and let-7 regulatory networks, as well as their interactions with multiple signaling pathways, additional downstream mechanisms beyond those examined here may contribute to the observed phenotypes. Future studies incorporating primary human RA FLS, in vivo intervention models, and comprehensive molecular analyses will be important to further clarify the role of this axis in RA pathogenesis.

## Conclusion

This study suggests that the LIN28A–let-7b regulatory axis may contribute to inflammatory and invasive activation of synoviocytes. By linking inflammatory stimulation to transcriptional programs controlling cytokine production and tissue remodeling, this pathway may represent a novel regulatory node in RA stromal pathology. Further investigation of this axis may provide new insights into mechanisms underlying persistent synovial inflammation and suggest potential therapeutic strategies targeting stromal cell activation in RA.

## Supplementary Information

Supplementary Material 1.

## Data Availability

No datasets were generated or analysed during the current study.
