# Supplementary material for: LIN28A‒let-7b axis drives the aggressive and proinflammatory phenotype of rheumatoid arthritis fibroblast-like synoviocytes
Source: Arthritis Res Ther. 2026 Apr 13;28:113. doi: 10.1186/s13075-026-03809-7 (PMC13188475; doi:10.1186/s13075-026-03809-7)

**Additional File**

**Table 1. Primers for quantitative PCR**

| Genes | Strand | Primer sequences | Origin |
| --- | --- | --- | --- |
| *Lin28a* | Forward  Reverse | 5´- GTTCGGCTTCCTGTCTATGA -3´  5´- GTTGTAGCACCTGTCTCCTT -3´ | Mouse |
| *Actb* | Forward  Reverse | 5´- TCTGGCACCACACCTTCTACA -3´  5´- TTTTCACGGTTGGCCTTAGG -3´ | Mouse |
| *LIN28A* | Forward  Reverse | 5´- GAGTGAGAGGCGGCCAAAA -3´  5´- TGATGATCTAGACCTCCACAGTTGTAG -3´ | Human |
| *IL6* | Forward  Reverse | 5´- AAAGAGGCACTGGCAGAAAA -3´  5´- TTTCACCAGGCAAGTCTCCT -3´ | Human |
| *IL8* | Forward  Reverse | 5´- ATGACTTCCAAGCTGGCCGTGGCT -3´  5´- TCTCAGCCCTCTTCAAAAACTTCT -3´ | Human |
| *MMP1* | Forward  Reverse | 5´- ATGAAGCAGCCCAGATGTGGAG -3´  5´- TGGTCCACATCTGCTCTTGGGA -3´ | Human |
| *MMP3* | Forward  Reverse | 5´- CACTCACAGACCTGACTCGGTT -3´  5´- AAGCAGGATCACAGTTGGCTGG -3´ | Human |
| *MMP9* | Forward  Reverse | 5´- CCCTCAGAGAATCGCCAGTACT -3´  5´- GCCACTACTTGTCCTTTGAGTC -3´ | Human |
| *TNFSF11* | Forward  Reverse | 5´- GCTTGAAGCTCAGCCTTTTG -3´  5´- CGAAAGCAAATGTTGGCATA -3´ | Human |
| *CXCL12* | Forward  Reverse | 5´- CATGCCGATTCTTCGAAAGC -3´  5´- TTCAGCCGGGCTACAATCTG -3´ | Human |
| *HMGA2* | Forward  Reverse | 5´- GAAGCCACTGGAGAAAAACGGC -3´  5´- GGCAGACTCTTGTGAGGATGTC -3´ | Human |
| *MCP1* | Forward  Reverse | 5´- CCCAAGCAGAAGTGGGTTCA -3´  5´- GCTGCAGATTCTTGGGTTGTG -3´ | Human |
| *ACTB* | Forward  Reverse | 5´- TGAGATGCGTTGTTACAGGAAGTC -3´  5´- GACTGGGCCATTCTCCTTAGAGA -3´ | Human |
| *Hmga2* | Forward  Reverse | 5´- AGAGGAAGACCCAAAGGCAGCA -3´  5´- GAGCAGGCTTCTTCTGAACGAC -3´ | Mouse |
| *Mmp1a* | Forward  Reverse | 5´- AGGAAGGCGATATTGTGCTCTCC -3´  5´- TGGCTGGAAAGTGTGAGCAAGC -3´ | Mouse |
| *Mmp1b* | Forward  Reverse | 5´- GCAGTTGTGGAAGATGCCATCG -3´  5´- CCATCAAATGTGTAGAGGTCACC -3´ | Mouse |
| *Mmp3* | Forward  Reverse | 5´- CACTCACAGACCTGACTCGGTT -3´  5´- AAGCAGGATCACAGTTGGCTGG -3´ | Mouse |
| *Mmp9* | Forward  Reverse | 5´- GGCTTTGCTGATGCTTCAGAA -3´  5´- CACAGCGTGGTGTTCGAATG -3´ | Mouse |
| *Il6* | Forward  Reverse | 5´- TTCCATCCAGTTGCCTTCTTG -3´  5´- GGGAGTGGTATCCTCTGTGAAGTC -3´ | Mouse |
| *Cxcl1* | Forward  Reverse | 5´- CTGGGATTCACCTCAAGAACATC -3´  5´- CAGGGTCAAGGCAAGCCTC -3´ | Mouse |
| *Cxcl2* | Forward  Reverse | 5´- ATGCCTGAAGACCCTGCCAAG -3´  5´- GGTCAGTTACCTTGCCTTTG -3´ | Mouse |
| *Ccl2* | Forward  Reverse | 5´- GCAGCAGCAGGTGTCCCAAAGAA -3´  5´- TGGTTCCGATCCAGGTTTTTA -3´ | Mouse |
| *Tnfsf11* | Forward  Reverse | 5´- GTGAAGACACACTACCTGACTCC -3´  5´- GCCACATCCAACCATGAGCCTT -3´ | Mouse |


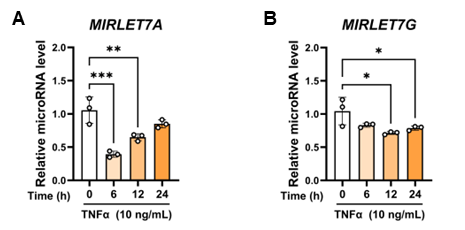


**Supplementary Figure S1. TNFα stimulation regulates the expression of let-7 family members in MH7A cells.**

MH7A cells were stimulation with TNFα (10 ng/mL) for the indicated time points, and the expression of let-7 family members was analyzed by qRT-PCR (n=3). (A) MIRLET7A expression levels in TNFα-stimulated MH7A cells. (B) MIRLET7G expression levels in TNFα-stimulated MH7A cells. Data are presented as mean ± SD, and statistical comparisons were performed using one-way ANOVA. *p<0.05, **p<0.01, ***p<0.001, and ****p<0.0001.


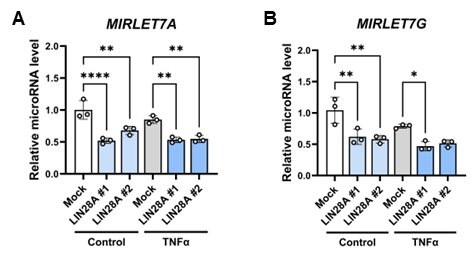


**Supplementary Figure S2. LIN28A overexpression modulates let-7 family expression in MH7A cells.**

MH7A cells were transfected with mock or LIN28A vector and cultured under basal conditions or stimulated with TNFα (10 ng/mL, 24 h). (A) MIRLET7A expression in mock and LIN28A-overexpressing MH7A cells. (B) MIRLET7G expression in mock and LIN28A-overexpressing MH7A cells. Data are presented as mean ± SD, and statistical comparisons were performed using one-way ANOVA. *p<0.05, **p<0.01, ***p<0.001, and ****p<0.0001.


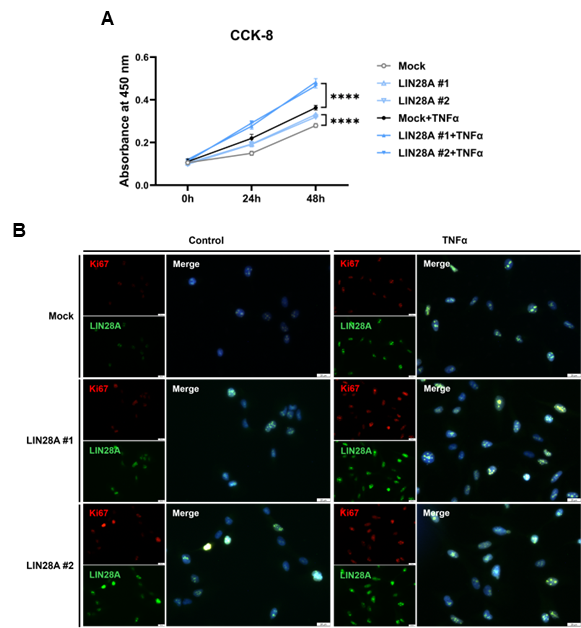


**Supplementary Figure S3. LIN28A overexpression enhances proliferation of MH7A cells.**

MH7A cells transfected with mock or LIN28A vector were cultured under basal conditions or stimulated with TNFα (10 ng/mL, 24 h). (A) Cell proliferation measured by CCK-8 assay (n=3). (B) Immunofluorescence staining of LIN28A and Ki67 in MH7A cells. Representative images are shown. Scale bar: 25 μm. Data are presented as mean ± SD, and statistical comparisons were performed using one-way ANOVA. ****p<0.0001.


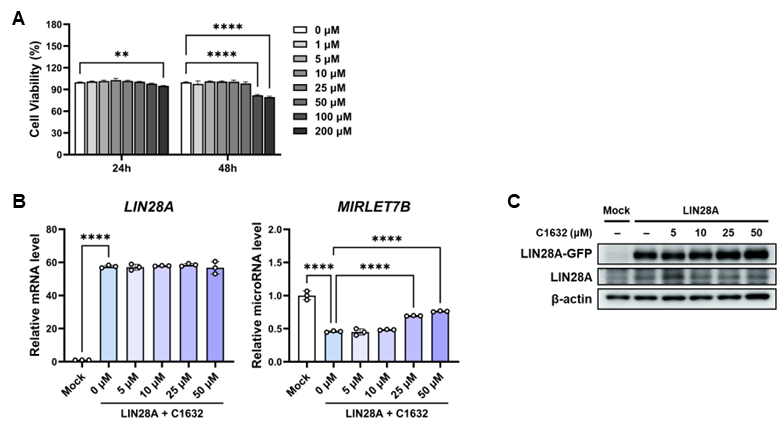


**Supplementary Figure S4. Determination of non-cytotoxic and biologically active concentrations of C1632.**

(A) MH7A cells were treated with increasing concentrations of C1632 (0–200 μM), and cell viability was assessed. No significant cytotoxicity was observed at concentrations up to 50 μM (n=3). (B) qRT-PCR analysis of LIN28A and MIRLET7B expression in LIN28A-overexpressing MH7A cells treated with non-cytotoxic concentrations of C1632 (0, 5, 10, 25, and 50 μM) for 24 h under basal conditions (n=3). (C) Western blot analysis of LIN28A protein expression following C1632 (0, 5, 10, 25, and 50 μM) treatment under the same conditions. Data are presented as mean ± SD, and statistical comparisons were performed using one-way ANOVA. **p<0.01 and ****p<0.0001.


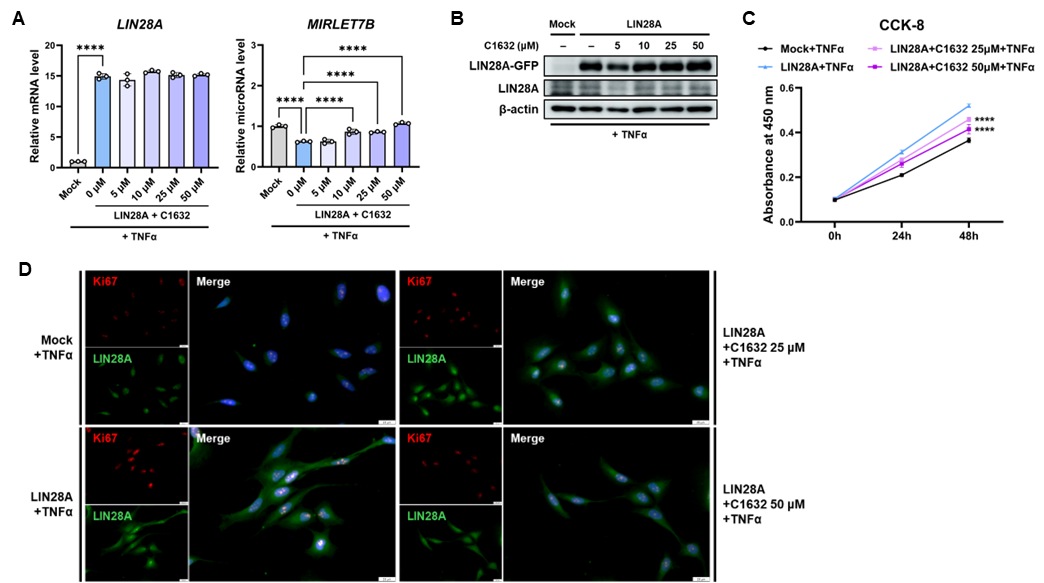


**Supplementary Figure S5. Effects of C1632 on LIN28A expression and cell proliferation in TNFα-stimulated MH7A cells.**

MH7A cells were pretreated with C1632 for 2 h followed by TNFα stimulation (10 ng/mL) for 24 h. (A) qRT-PCR analysis of LIN28A and MIRLET7B expression in MH7A cells treated with indicated concentrations (0, 5, 10, 25, and 50 μM) of C1632 (n=3). (B) Immunoblot analysis of LIN28A protein expression following C1632 (0, 5, 10, 25, and 50 μM) treatment (n=3). (C) Cell proliferation assessed by CCK-8 assay under basal conditions or TNFα stimulation in the presence of C1632 (25 or 50 μM) (n=3). (D) Immunofluorescence staining of LIN28A and Ki67 in MH7A cells under basal conditions or TNFα stimulation with C1632 (25 or 50 μM) treatment. Scale bar: 25 μm. Data are presented as mean ± SD, and statistical comparisons were performed using one-way ANOVA. ****p<0.0001.


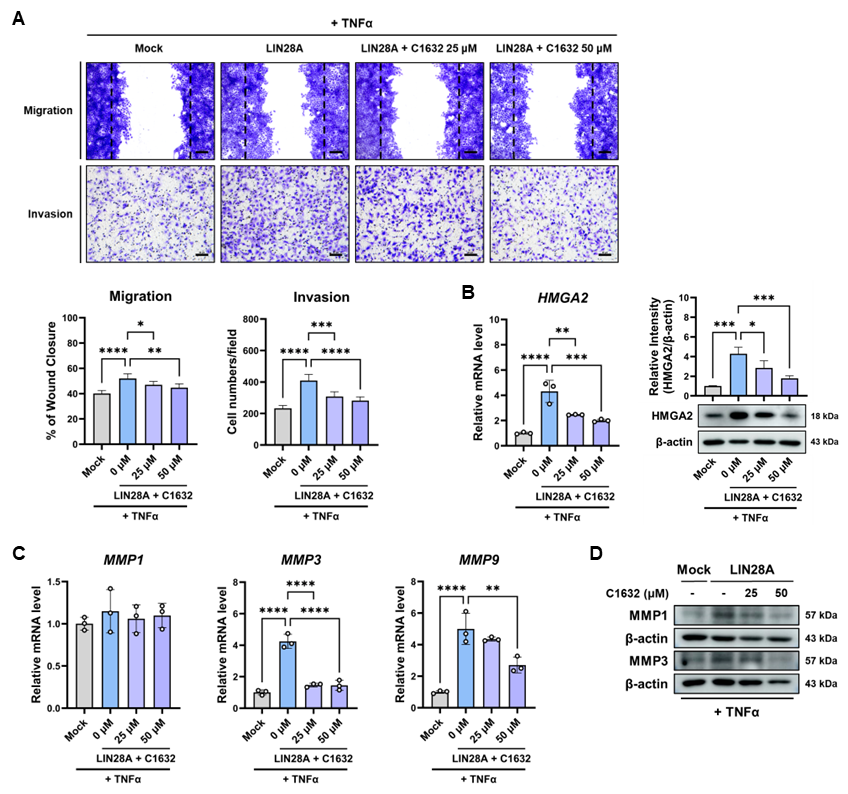


**Supplementary Figure S6. C1632 suppresses aggressive phenotypes in TNFα-stimulated MH7A cells.**

MH7A cells were pretreated with C1632 (25 or 50 μM) followed by TNFα stimulation (10 ng/mL, 24 h). (A) Representative images and quantification of wound healing and Matrigel invasion assays in MH7A cells treated with C1632 (n=5). Scale bars: 100 μm (migration) and 60 μm (invasion). (B) qRT-PCR and immunoblot analyses of HMGA2 expression following C1632 treatment (n=3). (C) qRT-PCR analysis of MMP1, MMP3, MMP9 expression in MH7A cells treated with C1632 (n=3). (D) Immunoblot analysis of MMP1 and MMP3 protein expression following C1632 treatment. Data are presented as mean ± SD, and statistical comparisons were performed using one-way ANOVA. *p<0.05, **p<0.01, ***p<0.001, and ****p<0.0001.


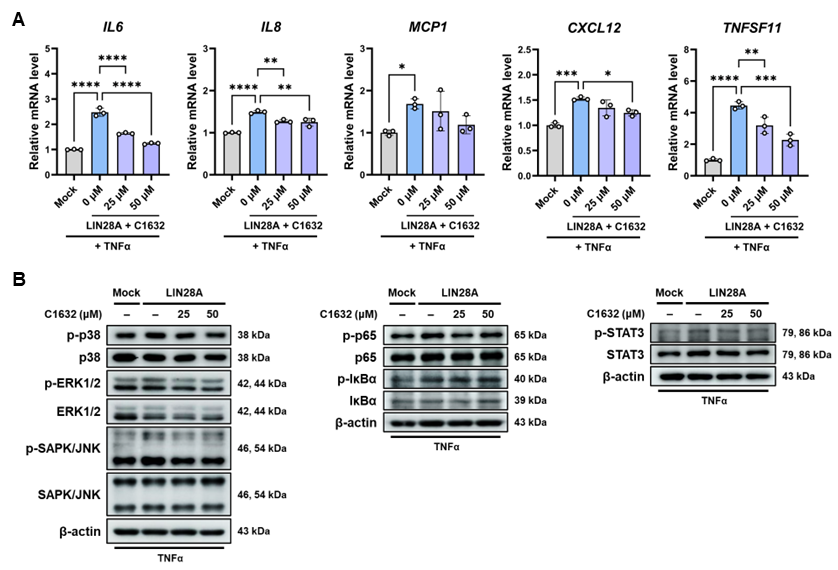


**Supplementary Figure S7. C1632 attenuates inflammatory cytokine expression and signaling pathway activation in MH7A cells.**

MH7A cells were pretreated with C1632 (25 or 50 μM) followed by TNFα stimulation (10 ng/mL, 24 h). (A) qRT-PCR analysis of inflammatory cytokine expression (IL6, IL8, MCP1, CXCL12, and TNFSF11) in MH7A cells treated with C1632 (n=3). (B) Immunoblot analysis of signaling pathway activation. Phosphorylation levels of p38 MAPK, ERK1/2, SAPK/JNK, NF-κB (p65), IκBα, and STAT3 were analyzed. Data are presented as mean ± SD, and statistical comparisons were performed using one-way ANOVA. *p<0.05, **p<0.01, ***p<0.001, and ****p<0.0001.

**Western Blot Images**

c
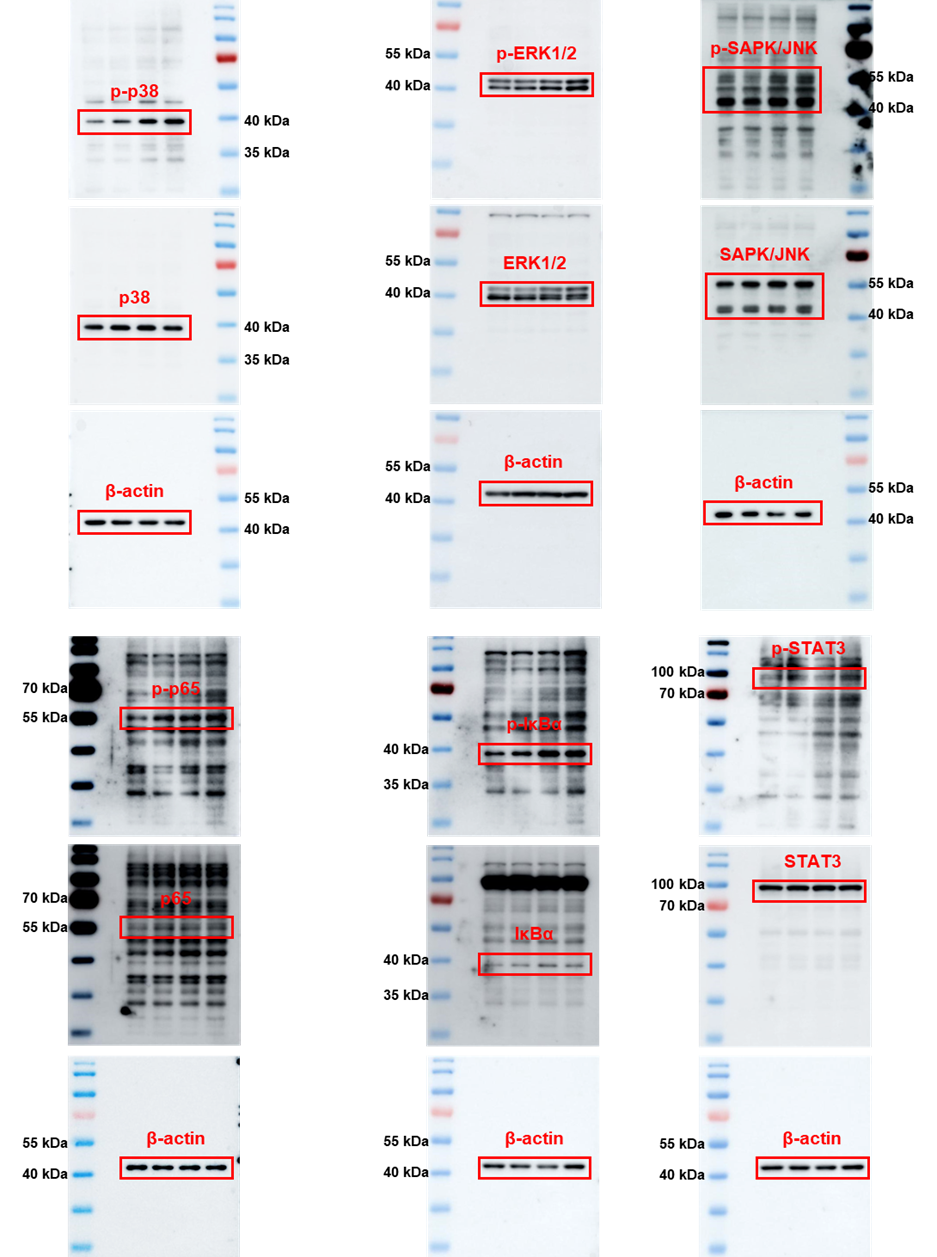


c
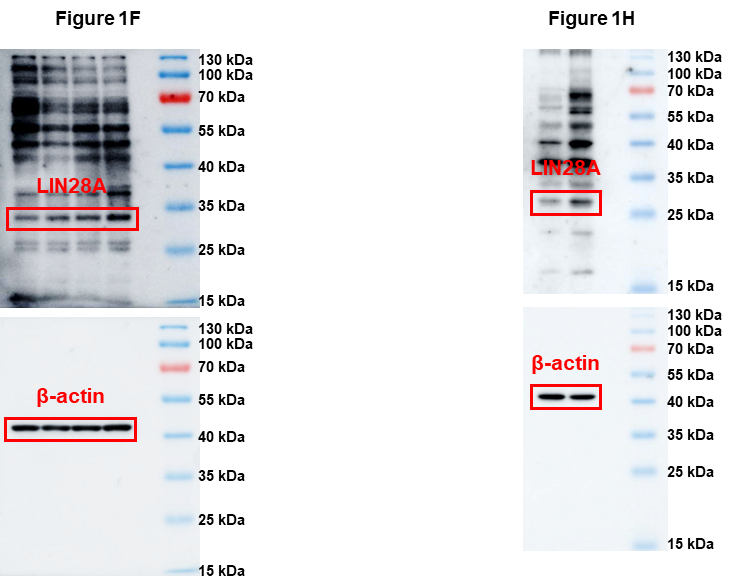


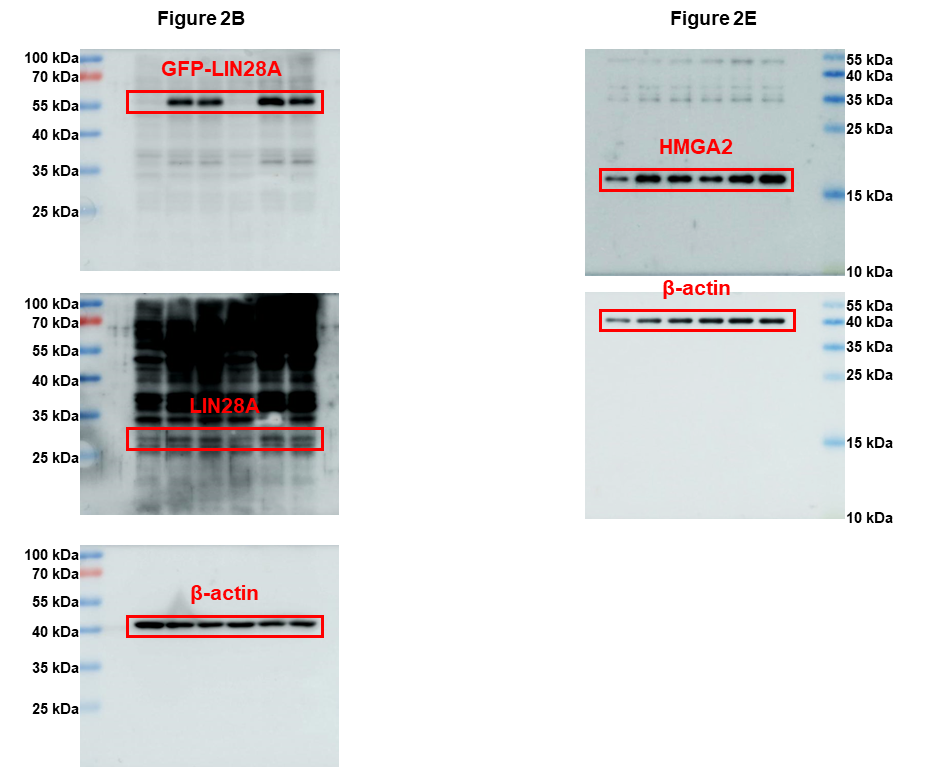


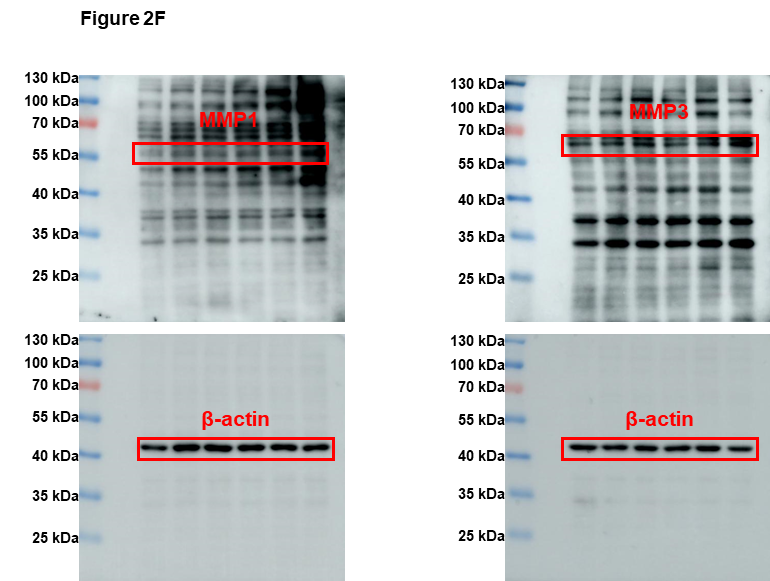


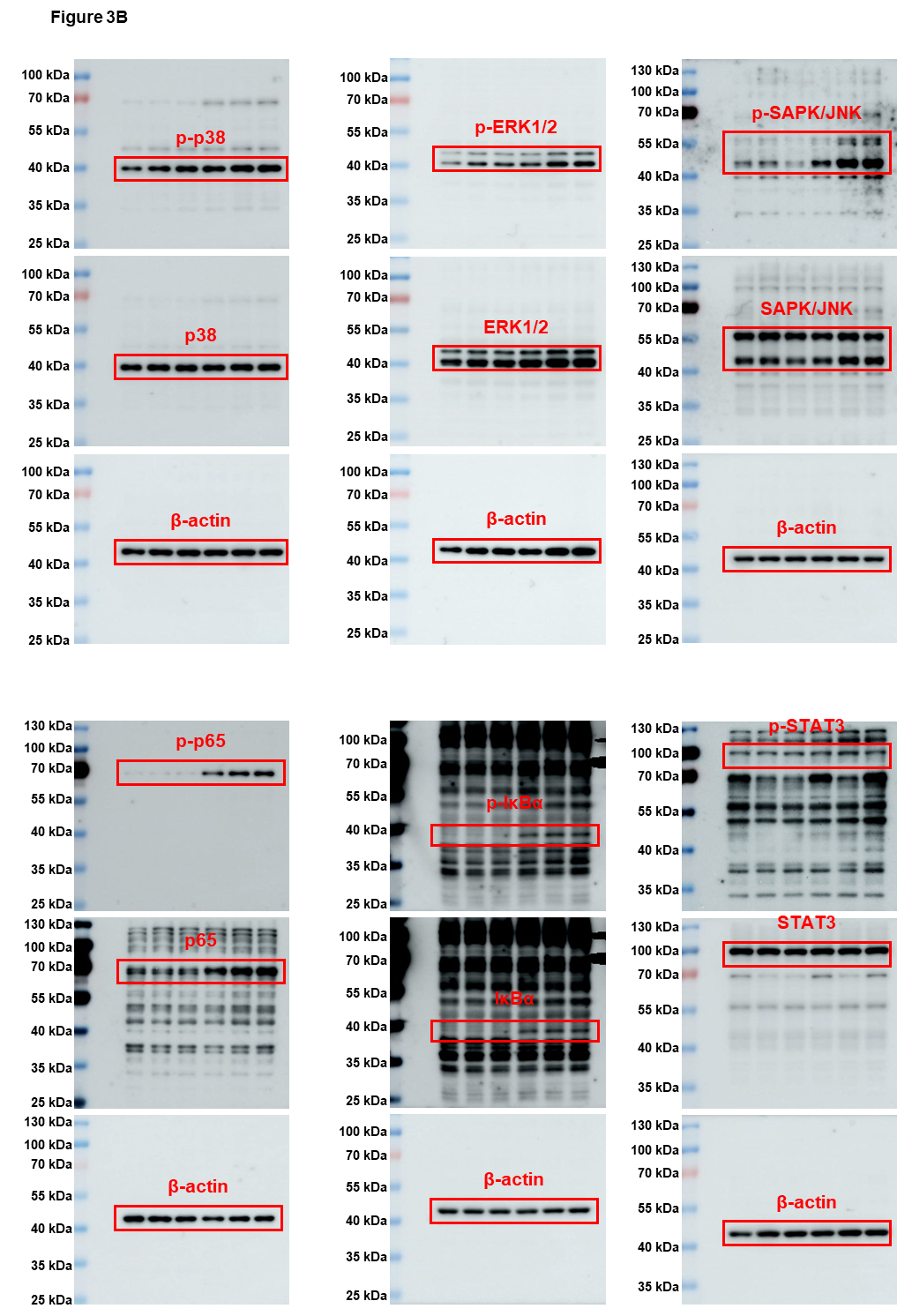


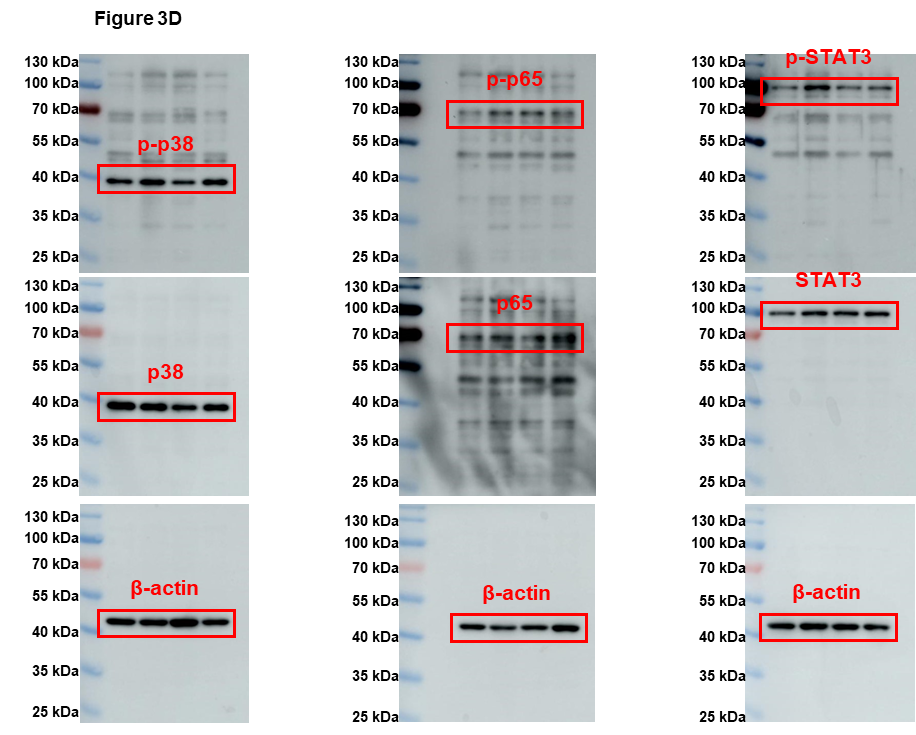


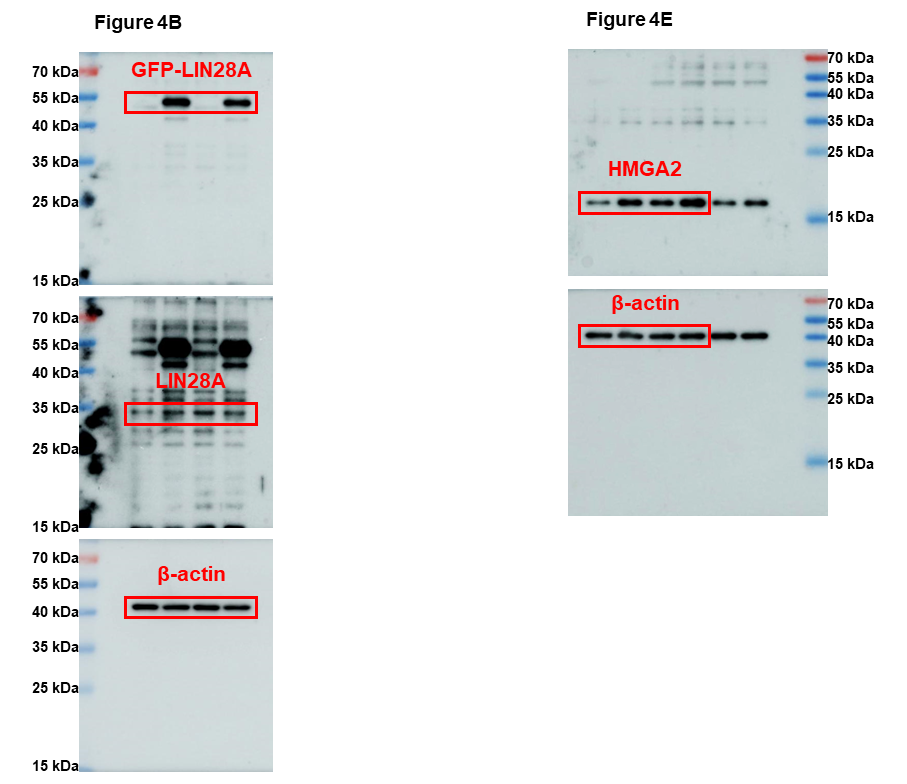


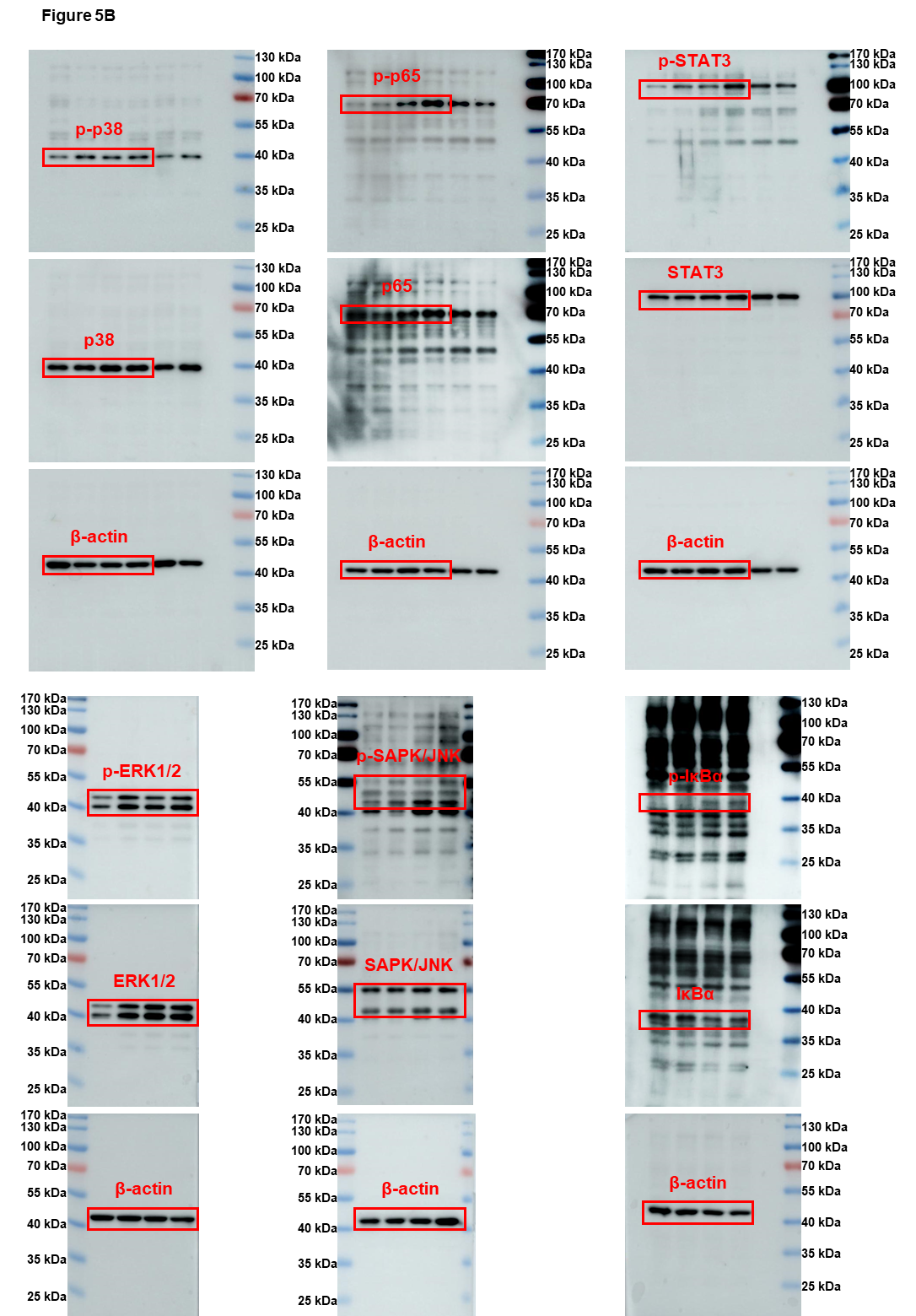


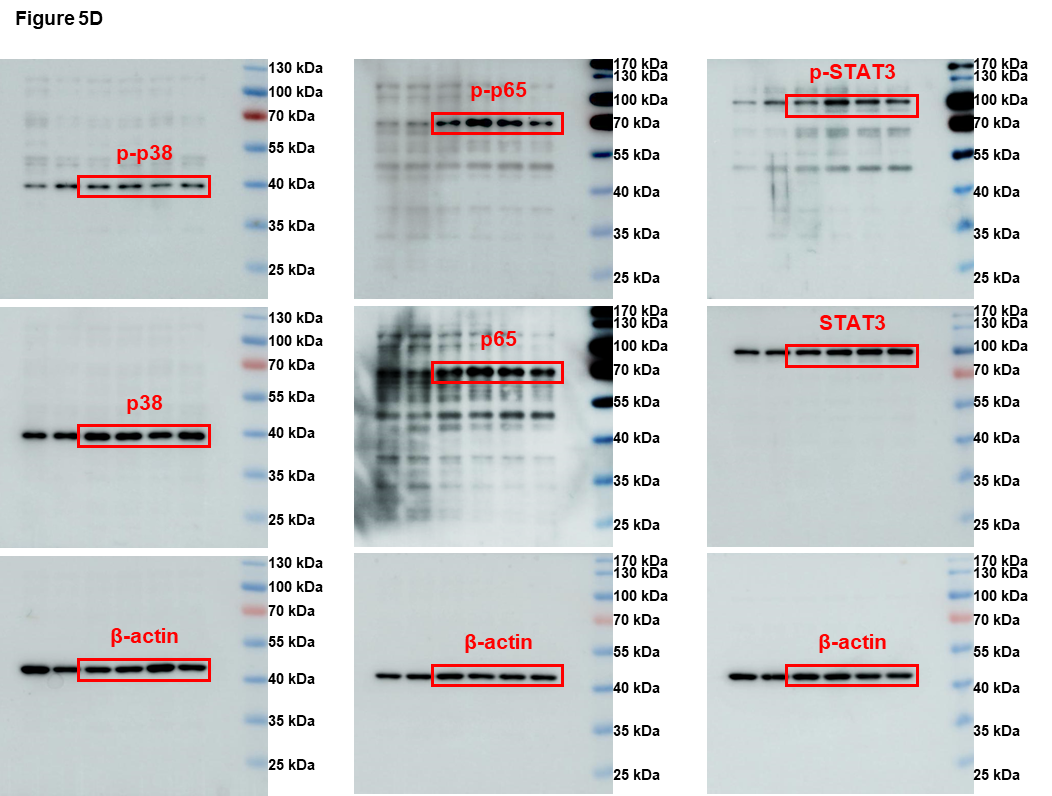


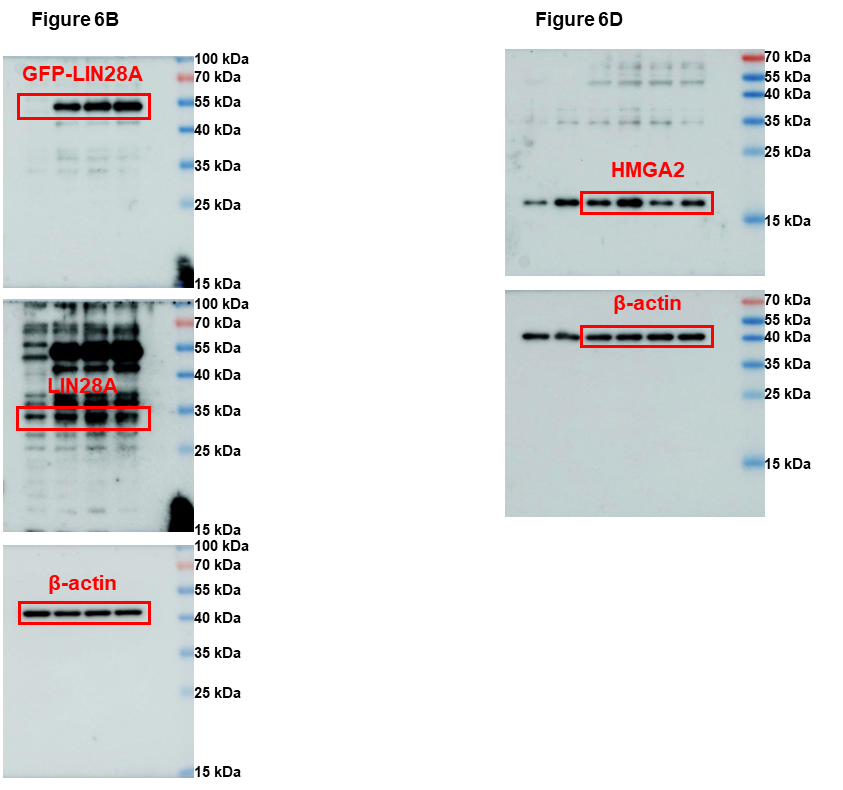


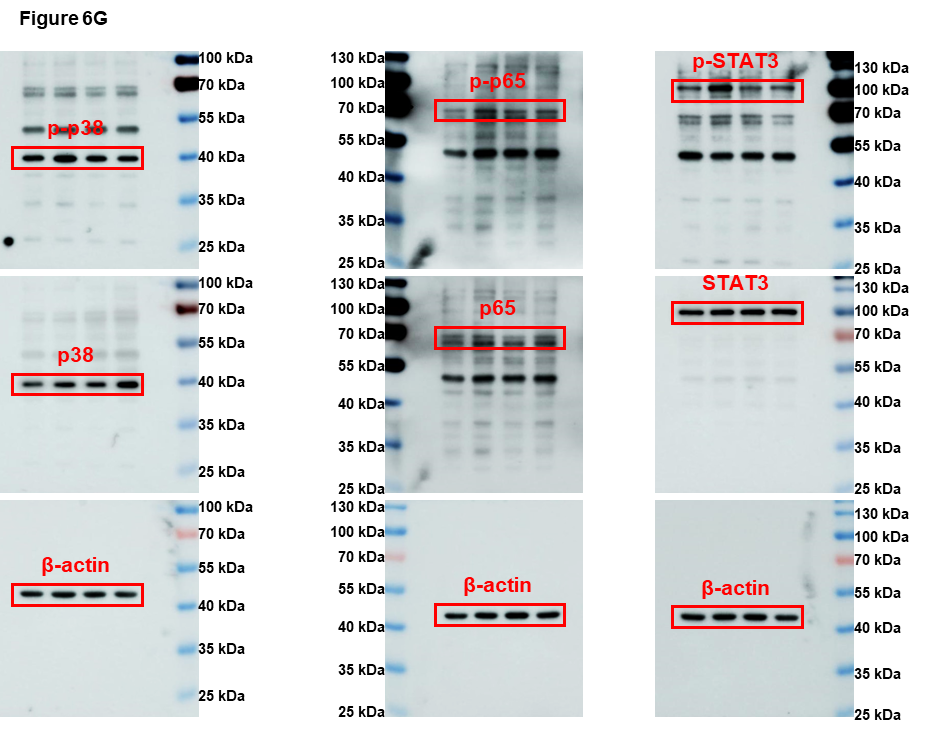


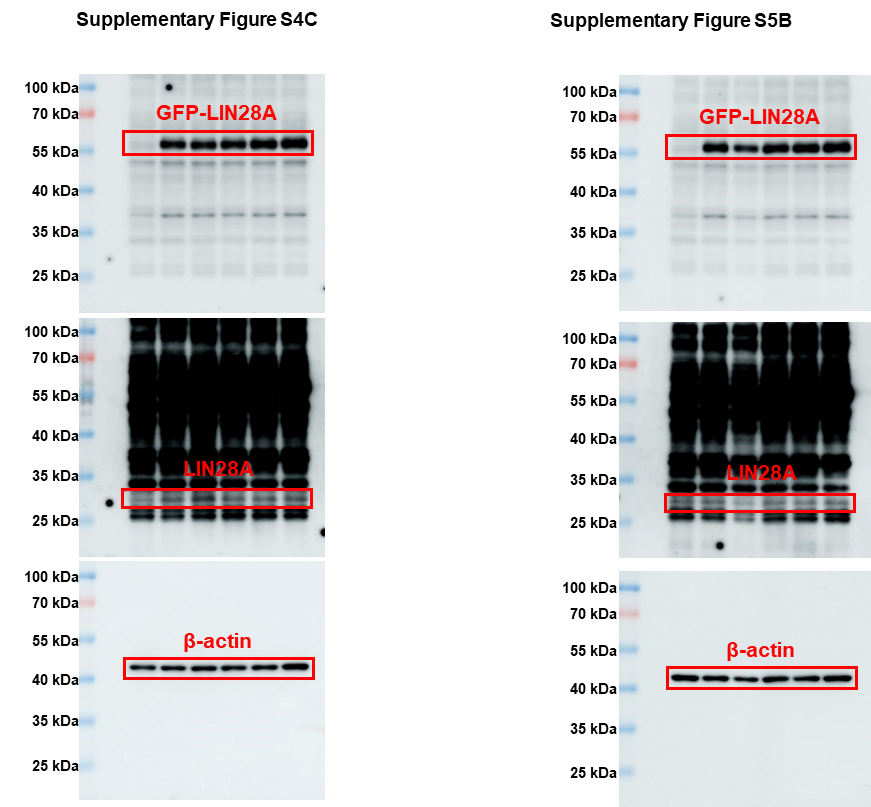


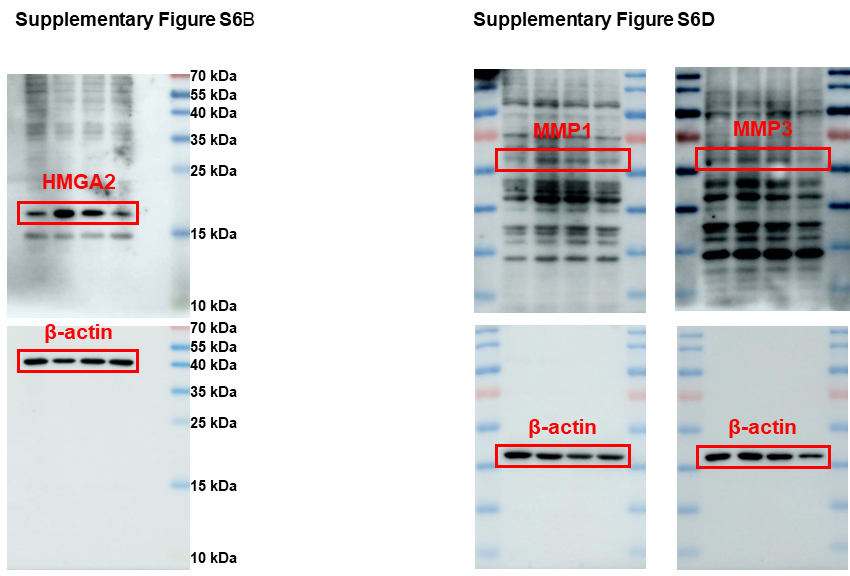


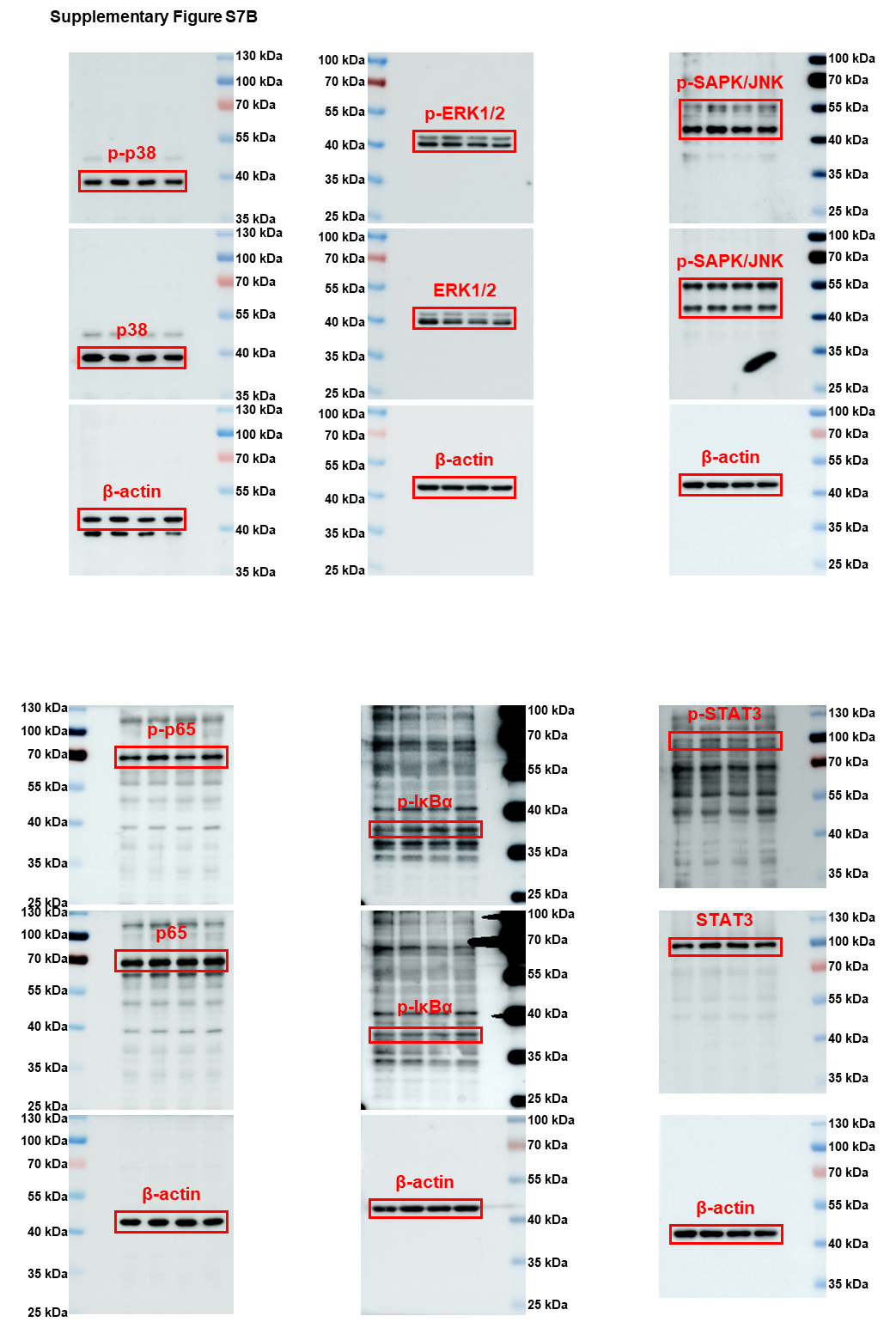

Supplement: Supplementary file 1 — Supplementary Material 1. [file 13075_2026_3809_MOESM1_ESM.docx]
